# Phosphatidylinositol-3-phosphate-dependent Klp98A recruitment regulates endosomal flux underlying developmental synaptic remodeling via Rab4

**DOI:** 10.1242/jcs.264782

**Published:** 2026-03-27

**Authors:** Kamaldeep Singh, Semanti Das, Dipti Rai, Sabyasachi Sutradhar, Asmita Sarkar, Jonathon Howard, Krishanu Ray

**Affiliations:** ^1^ Department of Biological Sciences, Tata Institute of Fundamental Research, Mumbai 400005, India; ^2^ Department of Molecular Biophysics and Biochemistry, Yale University, New Haven, CT 06520, USA

**Keywords:** Phosphatidylinositol-3-phosphate signaling, Synaptic plasticity, Axonal transport, Endosomes, Rab4, Klp98A, KIF16B, Vps34

## Abstract

The GTPase Rab4, which is essential for endosomal sorting and trafficking, is implicated in synaptic atrophy and dementia. To uncover the underlying mechanism, we studied the correlation between Rab4 vesicle transport in axons and episodic remodeling of synapses in the central nervous system (CNS) of *Drosophila* larvae. We found that synapse-bound traffic and presynaptic enrichment of Rab4 vesicles increase during the programmed transient contraction of gross synapse density in the ventral neuropil region at a specific larval stage. This reduction in the gross synapse density coincides with the episodic activation of insulin and Vps34-mediated signaling, which elevates phosphatidylinositol-3-phosphate levels on Rab4 vesicles. The presence of this phospholipid on Rab4-associated vesicles recruits a PX-domain-containing motor protein, Klp98A, accelerating their synapse-directed traffic. This, in turn, increases presynaptic enrichment of Rab4 during the developmentally programmed synapse contraction phase. Our findings elucidate the molecular mechanism that regulates developmental synaptic plasticity in the CNS via Vps34-depedent regulation of directed axonal transport of endosomes.

## Teaser

**Summary:** Elucidation of the molecular mechanism by which neuronal insulin signaling regulates axonal transport of recycling endosomes and promotes synaptic remodeling in the central nervous system of *Drosophila* larvae.

## INTRODUCTION

Rab4 (which has two forms, Rab4a and Rab4b, in mammals, and a single form in flies) is an organizer of endosomal sorting and plays a crucial role in several processes, such as recycling of surface receptors (van der Sluijs et al., 1992), axon outgrowth (Falk et al., 2014), maintenance of spine morphology and synaptic plasticity (Hoogenraad et al., 2010). Rab4 activation promotes its recruitment on the endosomal membrane and triggers vesicle trafficking through kinesin and dynein motors. Rab4-associated vesicles transport various cargoes, such as tetraspanins (Moretto et al., 2023), neuregulins (Ahmad et al., 2022), integrins (Shin et al., 2021) and astrotactins (Behesti et al., 2018), and regulate neuronal processes like trafficking and degradation of surface proteins, synapse formation, maturation and maintenance. Mutations in some of these cargoes are also implicated in neurodevelopmental (Behesti et al., 2018) and psychiatric disorders (Ahmad et al., 2022), implicating a potential role of Rab4-depdent trafficking in synaptic remodeling. Consistent with this conjecture, constitutive activation of Rab4 in neurons and its enrichment at the presynaptic terminals have been shown to induce synaptic atrophy in *Drosophila* (Dey et al., 2017). Furthermore, brain autopsies of individuals with Alzheimer's disease indicate an inverse correlation between the Rab4 levels in the cholinergic basal forebrain and CA1 neurons of the hippocampus, and their cognitive abilities (Ginsberg et al., 2011, 2010). Together, these observations indicate that Rab4 activation in neurons must be tightly controlled to maintain synaptic homeostasis. Therefore, understanding the yet unidentified molecular mechanisms regulating the Rab4 activation and the movement of Rab4-associated vesicles (henceforth called Rab4 vesicles) in neurons is essential for better comprehension of membrane trafficking functions in health and disease.

Besides the widely studied roles of insulin signaling in regulating metabolism (Tokarz et al., 2018), growth (Hakuno and Takahashi, 2018) and development (Dupont and Holzenberger, 2003), aberrant insulin signaling in the brain is also associated with cognitive decline with aging in *Drosophila* and humans (Augustin et al., 2017; Akintola and Van Heemst, 2015). Insulin signaling in the central nervous system (CNS) is implicated in amyloid precursor protein (APP) and huntingtin-associated protein 1 (HAP1) transport (Shieh et al., 2020) and regulates AMPA receptor endocytosis and synaptic plasticity in hippocampal neurons (Man et al., 2000). However, the underlying mechanisms remained unclear. In addition, a few studies have also shown that insulin regulates intracellular transport by modulating the recruitment of microtubule-based motor proteins in peripheral tissues (Imamura et al., 2003; Kumar et al., 2019). Specifically, insulin stimulation activates Rab4 and promotes its interaction with kinesin-2 to regulate GLUT4 (also known as SLC2A4) recycling in adipocytes (Imamura et al., 2003). Altogether, the evidence led us to postulate that insulin signaling might also regulate the axonal transport of Rab4 vesicles.

To test this hypothesis, we first established a developmental system of periodic synapse remodeling in the CNS of third-instar *Drosophila* larvae. This revealed that programmed contraction of synaptic content in the ventral neuromere coincides with episodic increase of Rab4 movement towards synapse and Rab4 enrichment at the presynaptic compartment. *In vivo* time-lapse imaging coupled with genetic and pharmacological perturbations showed that *Drosophila* Insulin-like peptide 2 (Ilp2, hereafter Dilp2) and *Drosophila* Insulin-like receptor (InR, hereafter dInR)-mediated insulin signaling could accelerate the anterograde axonal transport of a subset of Rab4 vesicles in *Drosophila* cholinergic neurons. Furthermore, a combined RNAi and chemical inhibitor screen of all known phosphoinositide 3-kinase (PI3Ks) in *Drosophila*, indicated a specific involvement of the class III PI3K Vps34 (also known as Pi3K59F in flies and PIK3C3 in mammals) in the process. We demonstrated that the activation of Vps34 by insulin signaling led to the production of phosphatidylinositol-3-phosphate [PI(3)P] on Rab4 vesicles in the axon. As a consequence, Klp98A (the homolog of mammalian KIF16B), a kinesin-3 family motor, is recruited by PI(3)P on Rab4 vesicles, activating their anterograde movement and elucidating a molecular basis for the acceleration of anterograde axonal transport of Rab4 vesicles in neurons by insulin signaling. Finally, we show that developmental upregulation of Klp98A recruitment on Rab4 vesicles in cholinergic neurons could reduce presynaptic contacts in the larval CNS. Altogether, our study (1) establishes an *in vivo* model system to investigate developmental synaptic plasticity in the CNS, (2) provides a molecular mechanism by which neuronal insulin signaling could regulate directional endosomal transport in the axons, and (3), elucidates functional consequences of altered endosomal traffic on synaptic homeostasis in the CNS.

## RESULTS

### The CNS of *Drosophila* undergoes synaptic remodeling during the third-instar larva stage

**Fig. 1.**
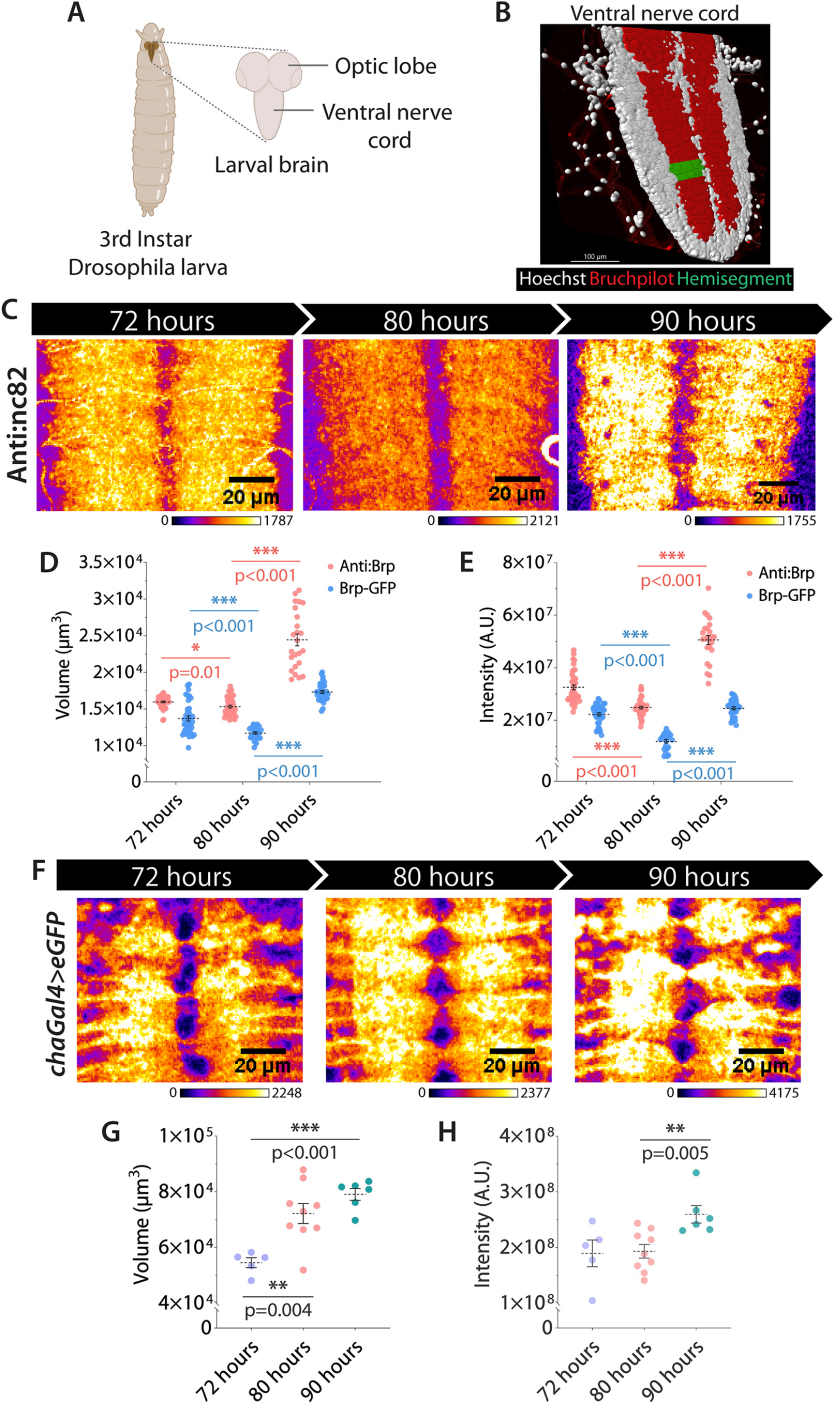
**Synaptic remodeling in the VNC during third-instar larval stage.** (A) Schematic illustrates different parts of the third-instar larval brain. Created in BioRender by Singh, K., 2025. https://BioRender.com/e3oqwsf. This figure was sublicensed under CC-BY 4.0 terms. (B) 3D volume rendered image of a larval VNC stained with anti-Bruchpilot (red, presynaptic junctions) and Hoechst 33342 (white, cortex). Green highlights a single neuromere hemisegment. Representative image of six repeats. (C) Representative images of A3–A6 region of larval VNC from 72–90 h AEL stained with anti-Bruchpilot (nc82) and presented from the look-up table used, which represents intensity in arbitrary units. (D,E) Mean±s.d. of the volume of the synaptic region (D) and Bruchpilot enrichment (intensity, E) in A3–A6 hemisegments marked by anti-Bruchpilot. (F) Representative images of A3–A6 segments of a larval VNC marked by *cha>eGFP* from 72–90 h AEL and presented from the look-up table used, which represents intensity in arbitrary units. (G,H) Mean±s.d. of GFP intensities and hemisegment volumes (I) and intensity (H) of cholinergic neuromeres marked by endogenous Bruchpilot–GFP. The pairwise significance of the difference is estimated using one-way ANOVA and Tukey's correction for multiple comparisons. A.U., arbitrary units.

At the third-instar larvae stage, *Drosophila* and their CNSs undergo a significant mass and volume expansion essential for metamorphosis (Vaufrey et al., 2018; Gan et al., 2014; Tyson et al., 2023), providing a suitable model system to investigate developmental synaptic plasticity and its underlying mechanism. The larval CNS (Fig. 1A) consists of two large optic lobes connected by a suboesophageal ganglion (SOG), and a bilaterally symmetric ventral nerve cord (VNC) composed of an easily distinguishable cortex region rich in cell bodies surrounding the synapse-bearing core called the neuropil (Fig. 1B). The VNC neuropil is divided into 12 segments (three thoracic and nine abdominal) along the anteroposterior body axis, with each segment comprising two bilaterally symmetric hemisegments. Each hemisegment consists of neurite arborizations and synapses contributed by segmentally organized sensory, motor and interneurons. It also contains neurite extensions form the anterior and posterior segments. Recent work has shown that *Drosophila* ventral nerve cord contains ∼45 million synapses, and single-cell transcriptomic dataset reveals that roughly half of these are cholinergic in nature (Azevedo et al., 2024; Allen et al., 2020).

Bruchpilot (Brp, an ELKS ortholog), a standard presynaptic marker of active chemical synapses (Rai et al., 2018; Wagh et al., 2006), was used to assess the synaptic density changes in the VNCs of developing larvae. The neuron-specific expression of fluorophore-tagged Brp-short (Brp-short–mCherry and Brp-short–mStraw) expressed through the chaGal4 driver produced supernumerary puncta in axons and VNC as overexpression artefacts, which were not observed in Brp immunostained or endogenous Brp–GFP expression backgrounds (Fig. S1A). Hence, we restricted our analysis to the global changes in chemical synapse organization using the latter two markers. Furthermore, we selected the A3–A6 hemisegments for these analyses due to negligible variation in Brp enrichment and the volume marked by Brp – henceforth referred to as ‘synaptic volume’ – among these hemisegments (Fig. S1B–D). The analysis identified two periods of synaptic remodeling during 72–90 h after egg laying (AEL). In the first period, between 72 and 80 h AEL, the Brp enrichment decreased significantly, although the synaptic volume marked by Brp immunostaining and endogenously expressed Brp–GFP decreased moderately (Fig. 1C–E; Fig. S1E). In the subsequent period, between 80 and 90 h AEL, both the synaptic volume and Brp enrichment increased significantly (Fig. 1C–E; Fig. S1E). Additionally, the volume occupied by cholinergic neurites, which constitutes a significant proportion of total neurite volume, estimated using cell-specific expression of soluble GFP in cholinergic neurons, increased from 72–90 h AEL (Fig. 1F–H). Altogether, the observations revealed a progressive increase in the volume occupied by cholinergic neurites and the chemical synapses in the neuropil region during the 72–90 h AEL period. Electron micrographs of the larval VNC at 72, 80 and 90 h AEL also corroborated with these findings (Fig. S1F), highlighting the existence of a programmed remodeling of synapses in the VNC during this period.

### Presynaptic Rab4 enrichment and synaptic density loss in developing CNS correlate with increased anterograde axonal transport of Rab4 vesicles

Previous work using ectopic expression of constitutively active and dominant-negative Rab4 mutants specifically in the cholinergic neurons has revealed an inverse correlation between presynaptic enrichment of active Rab4 and global synaptic volume in the third-instar larval CNS (Dey et al., 2017), suggesting that active Rab4 enrichment in the cholinergic neurons could abrogate significant synaptic contacts in *Drosophila* larval CNS. Given the high prevalence of cholinergic synapses in the larval CNS (Azevedo et al., 2024; Allen et al., 2020), these observations raised a possibility of a similar anti-correlation between developmentally regulated enrichment of Rab4 and Brp. Hence, we asked to what extent does a developmental regulation of Rab4 enrichment at the synaptic region of VNC and axonal transport of Rab4-associated vesicles in cholinergic neurons contribute to the observed synaptic remodeling in the CNS.

**Fig. 2.**
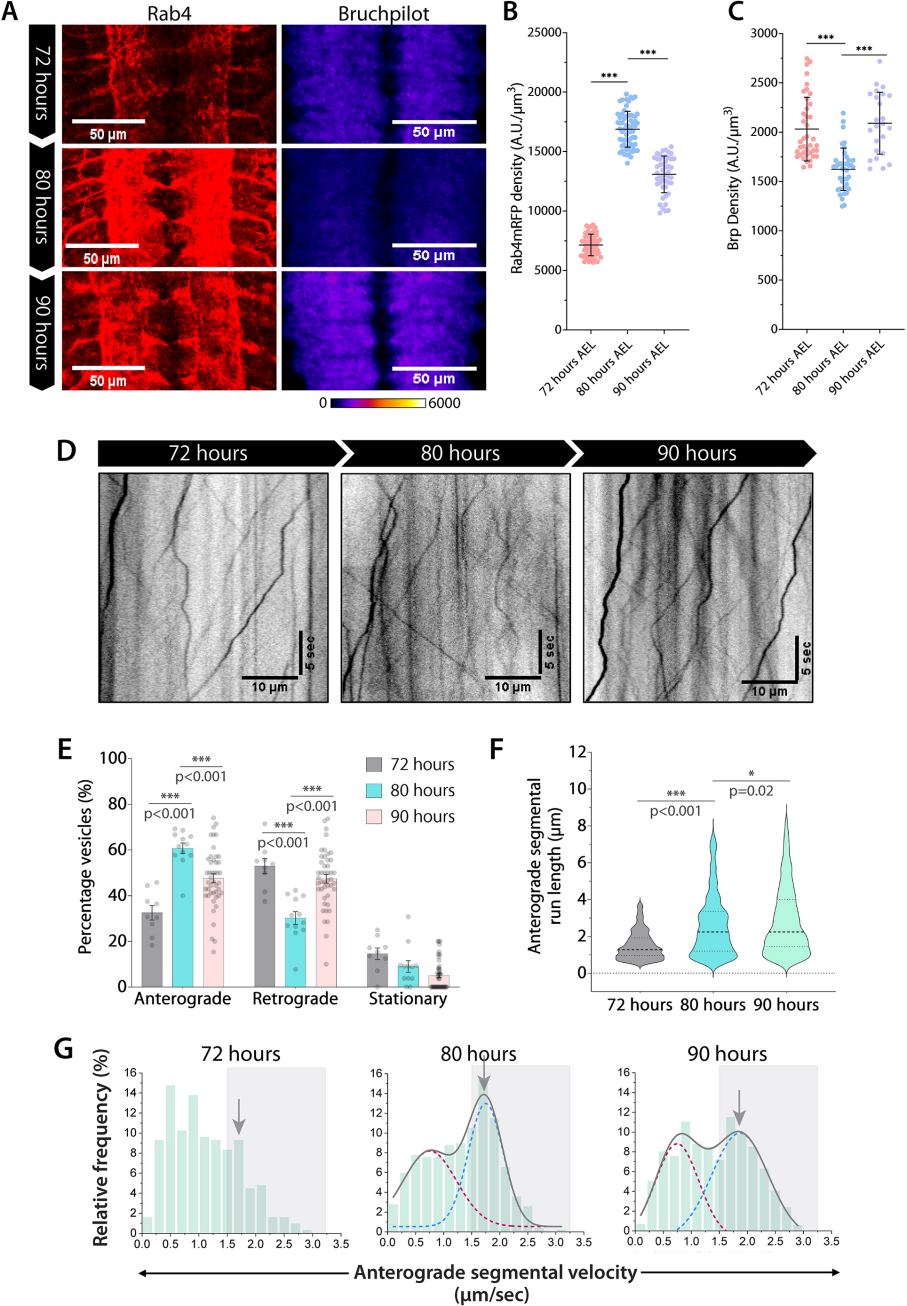
**Synapse enrichment and axonal transport of Rab4 vesicles in the developing larval VNC.** (A) A3–A6 segments of VNCs from 72–90 h AEL stained for Rab4 and Bruchpilot. (B,C) Rab4 (blue) and Bruchpilot (green) staining densities (A.U./μm^3^) in A3–A6 hemisegments (*n*>40, *N*=4–8 larvae). The density of Rab4 and Bruchpilot is statistically significantly different between 72 and 80 h and between 80 and 90 h AEL (Mann–Whitney *U*-test; ***P<0.001). Mean±s.d. (D) Representative kymographs of Rab4 vesicles transport at 72–90 h AEL. (E–G) Relative distribution of Rab4 vesicle movement (E, *n*≥9 segmental nerves, *N*=3–5 larvae), anterograde segmental run length (F, *n*>300 runs) and anterograde segmental velocity (G) of Rab4 vesicles. E is shown as mean±s.e.m., and F as violin plots with dashed lines highlighting median and quartiles. The cumulative distributions in G (gray) are shown as a sum of two Gaussians highlighting slow (maroon) and fast-moving (blue) populations (see Materials and Methods for details). The gray box marks the fast-moving (≥1.5 µm/s) runs. The arrow highlights peak values of fast-moving populations. The pairwise significance of difference was estimated using the Mann–Whitney *U*-test. A.U., arbitrary units.

In line with previous work, estimation of presynaptic Rab4 density and synapse density in the neuropil region revealed an expected antiphase correlation between episodic changes in the Rab4 accumulation and Brp levels during 72–90 h AEL (Fig. 2A,B). Furthermore, time-lapse imaging of distal axons of cholinergic neurons expressing Rab4–mRFP at 72, 80 and 90 h AEL (Fig. 2C; Fig. S2A, Movie 1) revealed a direct correlation between the anterograde flow of Rab4 vesicles and presynaptic Rab4 enrichment. The estimated fraction of anterogradely moving Rab4 vesicles (details in Materials and Methods) increased significantly from 72–80 h AEL and then decreased from 80–90 h AEL (Fig. 2D), with complementary changes in the retrograde fractions. The anterograde and retrograde fractions equalized at 90 h AEL. We reasoned that this programmed increase in the anterograde fraction of Rab4 vesicles from 72–80 h AEL could be promoted by: (1) enhanced anterograde velocity or processivity, (2) inhibition of retrograde movement, or (3) a combination of both.

To investigate the underlying cause, we analyzed the transport parameters of individual segmental runs, specifically run length and velocity, as per the procedure described in methods section. This revealed a significant increase in average anterograde segmental run length of Rab4 vesicles from 72–80 h AEL and a moderate increase from 80-90 h AEL (Fig. 2E). Additionally, anterograde velocity of Rab4 vesicles exhibited a bimodal distribution, categorized as the slower- (0.0–1.5 µm/s) and faster-moving (1.5–3.0 µm/s) segments (Fig. 2F). A similar bimodal distribution was also observed in the retrograde direction and was divided into slow (0.0–1.0 µm/s) and fast-moving (1.0–2.25 µm/s) runs (Fig. S2B). The frequency of fast-moving anterograde runs increased significantly from 72–80 h AEL followed by a significant reduction at 90 h AEL (Fig. 2F; Table S1; Kolmogorov–Smirnov test, *P*<0.001). We also observed a significant and progressive increase in the average retrograde segmental run length and velocity of Rab4 vesicles from 72–90 h AEL (Fig. S2B,C, Table S1; Kolmogorov–Smirnov test, *P*<0.001). Finally, although there was no significant change in the density of Rab4 vesicles in the axons from 72–80 h AEL, we observed a small but significant decrease in the density of Rab4 vesicles from 80 to 90 h AEL (Fig. S2D). The density could reduce due to a relatively higher retrograde flow or lowered biogenesis of Rab4 vesicles *in situ.*

Collectively, these results suggested that programmed alterations of the anterograde segmental velocity and run length of Rab4 vesicles during the developing third-instar larval stage could change the net anterograde flow of these vesicles in the axons, which in turn might alter the Rab4 enrichment and potentially cause the reduction in the synaptic density at the neuropil region. This observation also raised a new question – what triggers the increase in the anterograde speed and run-length of a fraction of Rab4 vesicles at 80 h AEL?

### Cell-autonomous insulin signaling can selectively increase the anterograde fraction of Rab4 vesicles

Acute insulin stimulation activates Rab4 via class-I PI3K, which recruits kinesin-2 and dynein on the Rab4-associated recycling endosome thereby increasing the GLUT4 receptor levels on the apical plasma membrane (Imamura et al., 2003; Shibata et al., 1997). A similar coincidence of Rab4, PI3K, and kinesin-2 motor activities are also implicated in the axonal transport of Rab4 vesicles in *Drosophila* (Dey et al., 2017). Also, previous work has reported insulin receptor (dInR) expression in sensory neurons (Li et al., 2022) and its enrichment in the axons of mechanosensory neurons of *Drosophila* pupa (Urwyler et al., 2019). Using a similar strategy as utilized before (Urwyler et al., 2019), we confirmed that dInR–CFP ectopically expressed in the cholinergic neurons could enrich along the length of the axons and in the cortex region of VNC (Fig. S3A–D). Therefore, we hypothesized that insulin signaling in cholinergic neurons could potentially regulate Rab4 vesicle movement in axons. To validate our results obtained from pharmacological perturbations, we knocked down dInR in the cholinergic neurons using two different UAS-InR-RNAi lines – BL31594 (InR^RNAi^-1, Valium1, weak) and BL51518 (InR^RNAi^-2, Valium 20, strong) – and confirmed the reduction in dInR RNA levels in cholinergic neurons (Fig. S3F). A similar relative difference in efficacies between these two RNAi lines was reported previously (Kanaoka et al., 2023). We chose to assess the effects of dInR knockdown and other perturbation experiments at 90 h AEL because the Rab4 vesicle movement parameters were intermediate between those seen at 72 and 80 h AEL at this stage, offering an optimal window to detect both activation and inhibition effects.

**Fig. 3.**
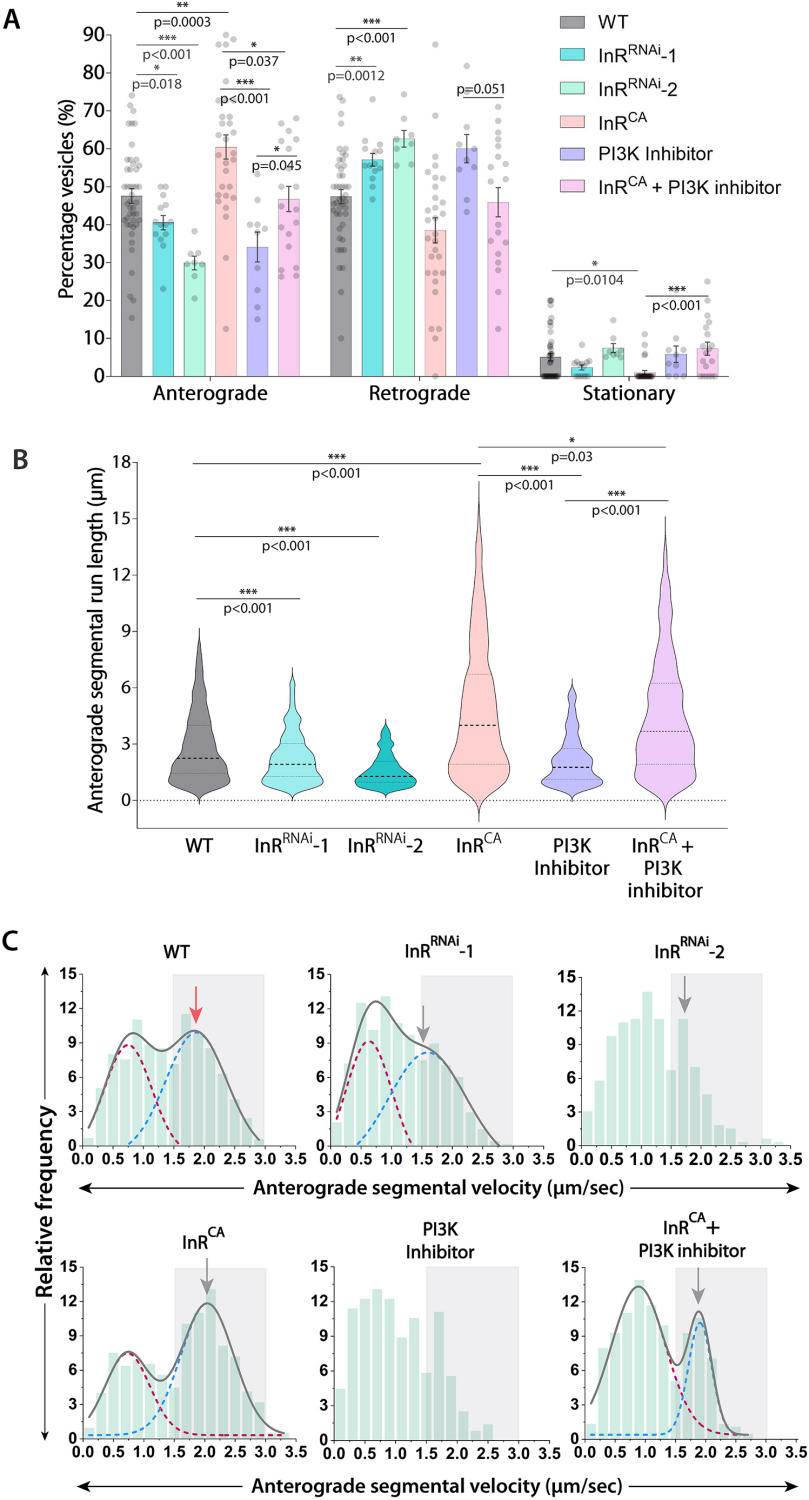
**InR signaling influences the axonal transport properties of Rab4 vesicles.** (A) Relative distribution of the Rab4 vesicle movement (*n*≥9 segmental nerves, *N*=3–5 larvae) in the wild-type control (WT), upon dInR^RNAi^, dInR^CA^ overexpression, and PI3K inhibitor backgrounds at 90 h AEL. Mean±s.e.m. (B,C) Anterograde segmental run length (B) and segmental velocity distribution (C) of Rab4 vesicles in different genetic and pharmacological backgrounds (*n*>200 runs). B is shown as violin plots with dashed lines highlighting median and quartiles. The cumulative distrubutions in C (gray) are shown as a sum of two Gaussians highlighting slow (maroon) and fast-moving (blue) populations. The gray box box marks the fast-moving (≥1.5 µm/s) runs. Gray arrows highlight peak values of fast-moving populations of treatment groups; red arrow highlights peak values of fast-moving populations of the control group. The pairwise significance of difference was estimated using the Mann–Whitney *U*-test.

The dInR knockdown significantly reduced the anterograde fraction of Rab4 vesicles by ∼8% (InR^RNAi^-1) and ∼18% (InR^RNAi^-2), respectively, at 90 h AEL (Fig. 3A; Fig. S3E, Movie 2). The differences in effective RNAi penetrance were consistent with the relative fold changes in the dInR mRNA levels observed in the two distinct RNAi backgrounds (Fig. S3F). As expected, the overexpression of a constitutively active insulin receptor (InR^CA^) variant in cholinergic neurons significantly increased the anterograde movement of Rab4 vesicles by ∼10% at 90 h AEL (Fig. 3A), matching the level observed at 80 h AEL (Fig. 2D). The increase in anterograde fraction upon overexpression of InR^CA^ was abolished by acute treatment with LY294002, which inhibits all known PI3Ks inside the cell (Fig. 3A). These data suggest that insulin signaling could act in a cell-autonomous manner, likely via PI3K, and the signaling dose could influence the anterograde movement of a subset of Rab4 vesicles in the axons.

Further analysis of the motility data also revealed a significant reduction in the anterograde segmental run length in both the InR^RNAi^ backgrounds and a significant increase in the InR^CA^ background (Fig. 3B). As expected, this dInR activity-dependent increase in anterograde runs was partially suppressed by acute PI3K inhibition in the InR^CA^ background (Fig. 3B). A comparatively milder effect was observed on the retrograde run length of Rab4 vesicles (Fig. S3G). Additionally, the frequency of fast-moving anterograde runs was reduced by different extents in both the InR^RNAi^-1 and -2 backgrounds and increased by ∼15% in the InR^CA^ overexpression background (∼15%; Fig. 3C; Table S1). As expected, the LY294002 treatment in the InR^CA^ background suppressed the anterograde runs by ∼14% as compared to the untreated InR^CA^ preparations (Fig. 3C; Table S1), indicating that the enhancement was likely to be caused due to ectopic dInR activation. Similar but relatively milder effects were observed on the frequency of fast-moving retrograde runs in the InR^RNAi^-1, InR^CA^ and LY294002-treated InR^CA^ backgrounds (Fig. S3H; Table S1). Such a coupled change has been reported both *in vivo* and *in vitro* due to constitutive association between kinesin and dynein motors (Pilling et al., 2006; Hendricks et al., 2010).

Altogether, these observations indicate that the activation of cell-autonomous insulin signaling via dInR could increase the anterograde speed and processivity of Rab4 vesicles leading to a significant elevation of the anterograde flow of these vesicles towards the synapse (Fig. 2D). However, its effects on the retrograde movement of Rab4 vesicles were less pronounced. This asymmetric impact of changes in the InR-dependent signaling caused significant shifts in the Rab4 vesicle density in the axons, which were markedly elevated in the InR^RNAi^ backgrounds (Fig. S3I), possibly due to reduced anterograde flow, and marginally reduced in the InR^CA^ background. Notably, this trend was reversed upon PI3K inhibition (Fig. S3I), implying that insulin signaling could modulate Rab4 vesicle trafficking in axons. These findings further highlight that perturbations in insulin signaling can also influence the biogenesis or turnover of Rab4 vesicles, potentially impacting endosomal dynamics during synaptic remodeling.

### Treatment with human insulin and Dilp2 increases the anterograde fraction, velocity and run length of Rab4 vesicles

**Fig. 4.**
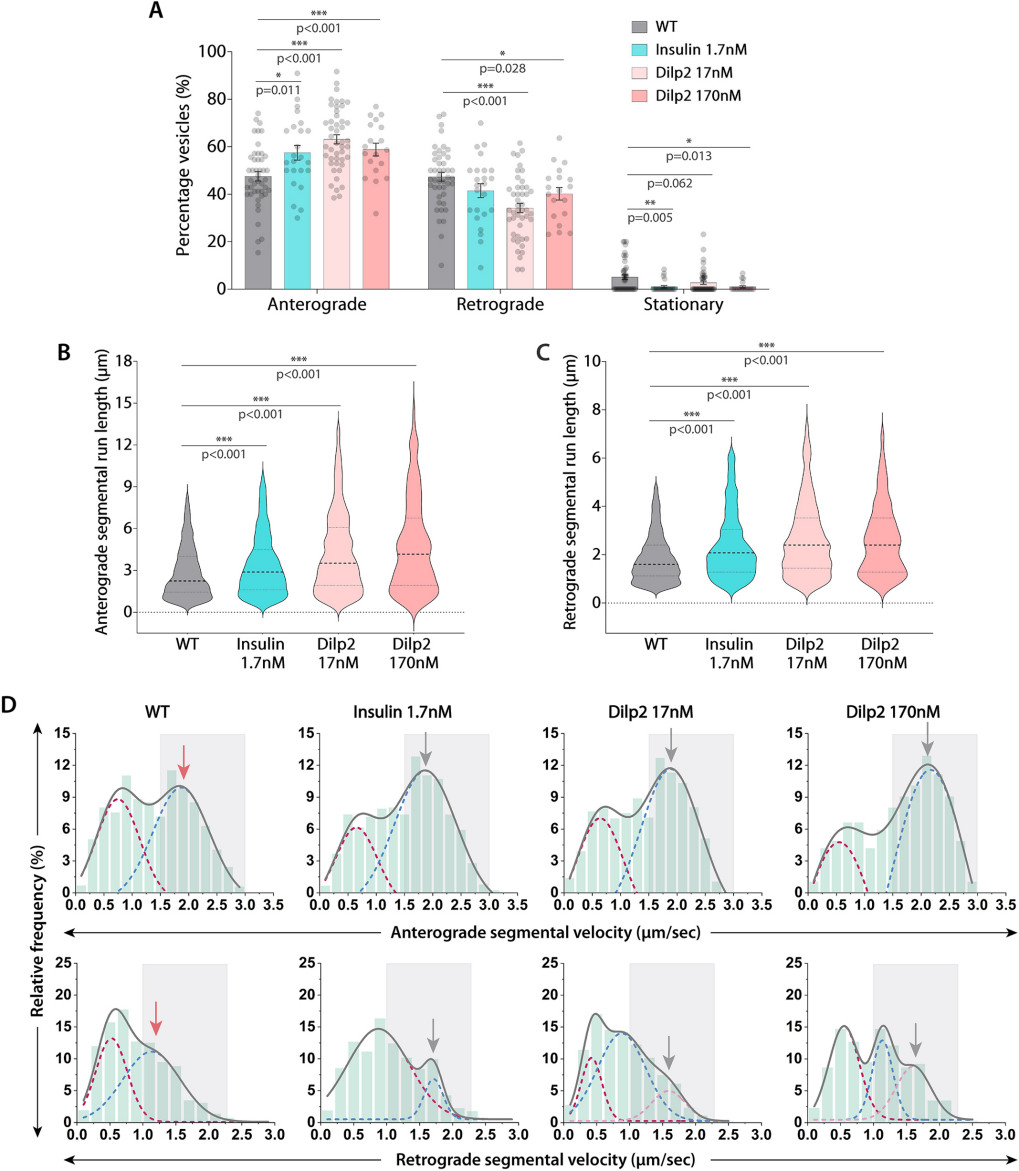
**Acute stimulation with human insulin and Dilp2 increases the anterograde fraction, velocity and run length of Rab4 vesicles in axons.** (A) Relative distribution of the Rab4 vesicle movement (*n*≥19 segmental nerves, *N*=3–5 larvae) in the wild-type control without insulin (WT) and in the presence of different concentrations of human insulin and Dilp2 at 90 h AEL. Mean±s.e.m. (B,C) The anterograde (B) and retrograde (C) segmental run length (µm) of Rab4 vesicles in the wild-type control (WT) and different treatment backgrounds (*n*>300 runs) at 90 h AEL. Results are shown as violin plots with dashed lines highlighting median and quartiles. The pairwise significance of difference was estimated using the Mann–Whitney *U*-test. (D) Relative distribution of anterograde (top row) and retrograde (bottom row) segmental velocity of Rab4 vesicles in the wild-type control (WT) and different treatment backgrounds at 90 h AEL. The cumulative distributions (gray) are shown as a sum of multiple Gaussians to highlight slow (maroon) and fast-moving (blue and pink) populations. Gray box marks the fast-moving runs (≥1.5 µm/s for anterograde and ≥1.0 µm/s for retrograde). Gray arrows highlight peak values of fast-moving populations of treatment groups; red arrows highlight peak values of fast-moving populations of the control group.

To understand how insulin signaling could increase the anterograde velocity of Rab4 vesicles in real time, we developed a pharmacological paradigm to stimulate the process in axons. Acute stimulation (15 min) with 1.7 nM human insulin in the bath (Movie 3) significantly increased the proportion of anterogradely moving Rab4 vesicles by ∼10% and proportionately reduced the retrograde fraction (Fig. 4A). Of the eight *Drosophila* insulin-like peptides (Dilps) (Nässel and Broeck, 2016), Dilp2 and Dilp5 are the most abundantly expressed in the brain tissue (Semaniuk et al., 2021), with Dilp2 having the highest conservation (∼35% sequence identity) with human insulin (Brogiolo et al., 2001). Therefore, to understand the physiological relevance of the effect, we tested the effects of recombinant Dilp2 and Dilp5 treatment on the axonal transport of Rab4 vesicles. Like human insulin, acute stimulation with 17 nM and 170 nM Dilp2 also increased the net anterograde fraction of Rab4 vesicles (Fig. 4A). However, acute stimulation with Dilp5 even at 170 nm had no significant effect on the anterograde fraction of Rab4 vesicles, indicating the specificity of Dilp2 action (Fig. S4A; Table S1). These observations suggest that both human insulin and Dilp2 stimulation can selectively increase the anterograde flow of Rab4 vesicles in *Drosophila* axons.

Subsequent detailed analysis of the movement further suggested that Dilp2 and human insulin treatment could significantly increase the average segmental run length and frequency of fast-moving runs in the anterograde direction (Fig. 4B,D; Table S1; Kolmogorov–Smirnov test, *P*<0.001). They also increased the average segmental run length and velocity in the retrograde direction to a comparatively lesser extent (Fig. 4C,D; Table S1). Although Dilp5 treatment did not affect the anterograde segmental run-length, the frequency of fast-moving anterograde runs was slightly reduced at higher concentrations (Fig. S4B,D; Table S1). Additionally, there was a moderate but significant increase in segmental run length along with a significant decrease in frequency of fast-moving runs in the retrograde direction (Fig. S4C,D, Table S1; Kolmogorov–Smirnov test, *P*<0.001). Finally, although the density of particles remained unaltered upon treatment with 1.7 nM insulin and 17–170 nM Dilp2, it increased significantly upon treatment with 17 nM Dilp5 (Fig. S4E), indicating a potential increase in Rab4 vesicles biogenesis *in situ* due to Dilp5 stimulation. The observation also separated the insulin-dependent changes in the movement characteristics from the biogenesis of Rab4 vesicles.

Together, these observations establish that acute stimulation with human insulin and Dilp2 can preferentially enhance the anterograde motility of a subset of Rab4 vesicles in a manner similar to that of constitutive dInR activation in cholinergic neurons. The effects occur at a relatively fast time scale and human insulin was more effective, likely owing to its enhanced stability. Thus, establishing this pharmacological assay system allowed us to further probe the fast-acting downstream pathways, effectors and molecular mechanisms involved in accelerating the transport of a subset of Rab4 vesicles upon insulin stimulation.

### Vps34 activity is required for stimulating the anterograde movement of Rab4 vesicles downstream of insulin signaling

Next, to identify the molecular basis of the insulin-stimulated acceleration of Rab4 vesicles, we probed the functions of PI3Ks, which lies immediately downstream of dInR. A type I PI3K has been shown to regulate Rab4 activation and endosomal turnover in adipocytes and neurons (Dey et al., 2017; Imamura et al., 2003; Chamberlain et al., 2010), and the above data further implicated PI3K activity downstream of InR-dependent activation of Rab4 vesicle movements in axons (Fig. 3A). *Drosophila* sensory neurons express three different classes of PI3Ks (Li et al., 2022; Brown et al., 2014), each with distinct subcellular localization pattern (Di Paolo and De Camilli, 2006). We conducted a combinatorial screen using *in vivo* live imaging of Rab4 vesicles in the presence of class-specific chemical inhibitors and tissue-specific knockdown of all three PI3Ks (Movies 4, 5).

**Fig. 5.**
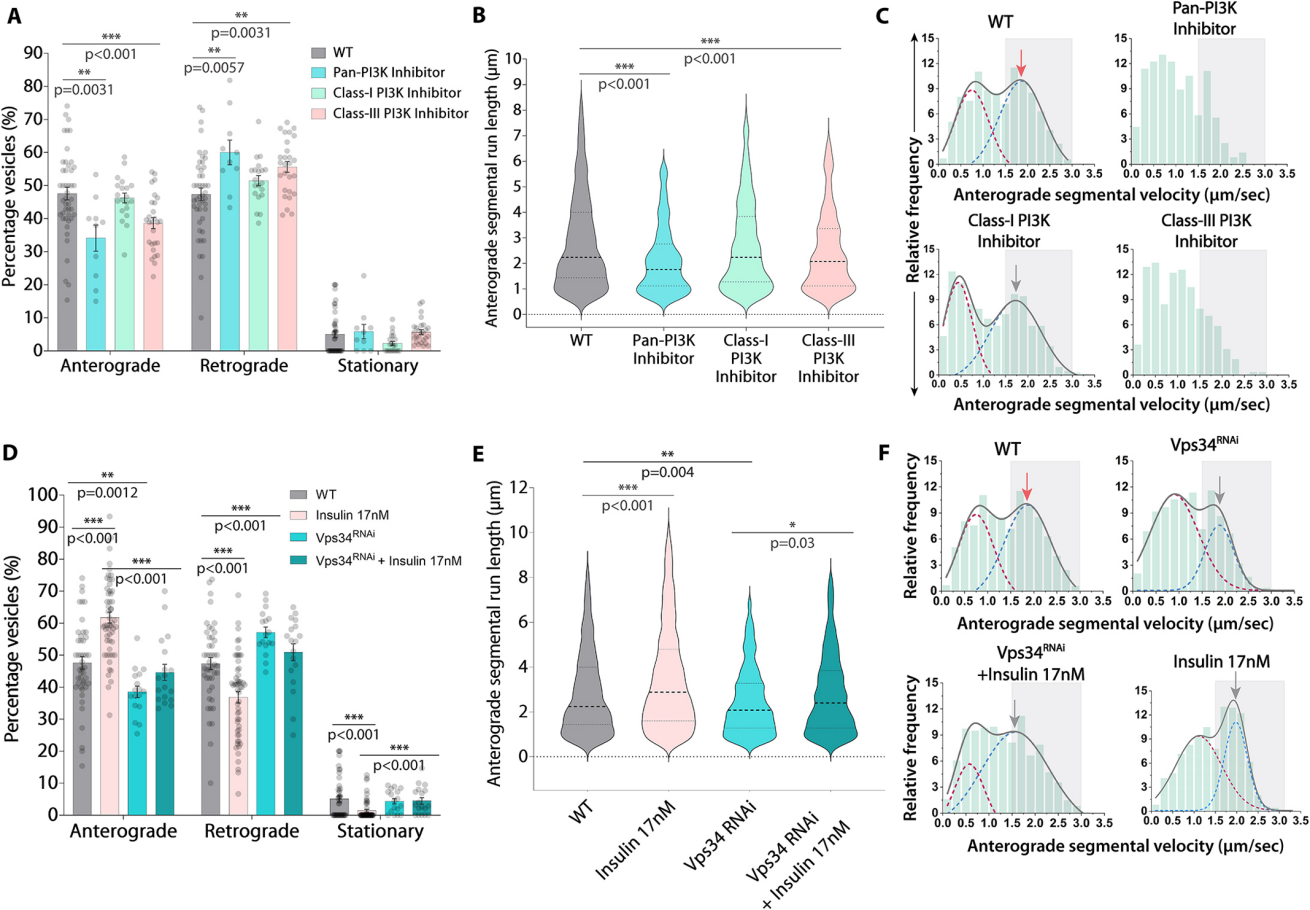
**Acute inhibition and knockdown of the class III PI3K Vps34 reduces anterograde fraction, run length and velocity of Rab4 vesicles in axon.** (A–C) Relative distribution of movement (A, *n*>10 segmental nerves, *N*=3–5 larvae), anterograde segmental run length (B, *n*>800 runs) and anterograde segmental velocity distributions (C) of Rab4 vesicles in the wild-type control (WT) and in the presence of different class-specific PI3K inhibitors at 90 h AEL. The cumulative distributions in C (gray) are shown as a sum of two Gaussians highlighting slow (maroon) and fast-moving (blue) populations. Gray box marks the fast-moving (≥1.5 µm/s) runs. (D–F) The relative distribution of the Rab4 vesicle movement (D, *n*≥17 segmental nerves, *N*=3–5 larvae), anterograde segmental run length (E, *n*>700 runs), anterograde segmental velocity distributions (F) in the wild-type control (WT) and Vps34^RNAi^ background in the absence and presence of insulin at 90 h AEL. The cumulative distribution in F (gray) is shown as as a sum of two Gaussians highlights slow (maroon) and fast-moving (blue) populations and the gray box marks the fast-moving (≥1.5 µm/s) runs. A and D are shown as mean±s.e.m., and B and E as violin plots with dashed lines highlighting median and quartiles. The pairwise significance of difference was estimated using the Mann–Whitney *U*-test. Gray arrows highlight peak values of fast-moving populations of treatment groups; red arrows highlight peak values of fast-moving populations of the control group.

Acute inhibition (15 min) of all classes of PI3Ks using a pan-PI3K inhibitor (LY294002) significantly reduced the anterograde runs (by ∼14%), average segmental run length, and the frequency of fast-moving anterograde runs (∼22%; Fig. 5A–C, Table S1; Kolmogorov–Smirnov test, *P*<0.001) of Rab4 vesicles. Likewise, acute inhibition of Class III PI3K (Vps34) using SAR405 led to a similar effect (Fig. 5A–C; Table S1), thereby indicating a possibly direct role of Vps34 in this process. In contrast, acute inhibition of the class-I PI3K using HS173 did not affect the anterograde fraction (Fig. 5A), although it significantly altered the frequency of fast-moving runs (Fig. 5C; Table S1; Kolmogorov–Smirnov test, *P*<0.01). Consistent with the effect of insulin treatment and perturbations of insulin signaling (Fig. 4, Figs S3 and S4), we observed a relatively small change in the retrograde transport parameters after PI3K inhibitor treatments (Fig. S5). As a class II PI3K inhibitor is not commercially available and the effects of Vps34 inhibitor treatment largely phenocopied the effects observed with pan-PI3K inhibitor treatment, these findings suggested that Vps34 activity could selectively regulate the anterograde fraction of Rab4 vesicles in axons.

To confirm the conjecture, we individually knocked down each one of the three PI3Ks in cholinergic neurons using established UAS-RNAi lines. It revealed that only Vps34 RNAi could significantly reduce the anterograde fraction by nearly 10% (Fig. 5D; Fig. S6), as well as the run length (Fig. 5E), and the frequency of fast-moving runs of Rab4 vesicles (∼11%; Fig. 5F and Table S1; Kolmogorov–Smirnov test, *P*<0.001). Furthermore, acute insulin stimulation in the Vps34 RNAi background failed to increase the anterograde fraction of Rab4 vesicles to the expected level (Fig. 5D) and only marginally improved the run length (Fig. 5E) and frequency of fast-moving anterograde runs (∼4%; Fig. 5F).

In comparison, RNAi against the PI3KC1 (class I) catalytic subunit had no significant effect on the anterograde fraction, segmental run length and the frequency of fast-moving anterograde runs (Fig. S6A,B,D). Although RNAi of the PI3KC1-regulatory subunit marginally decreased the frequency of fast-moving anterograde runs and significantly increased both the anterograde and retrograde run length (Fig. S6), it had no significant impact on the overall movements (Fig. S6C). RNAi against PI3KC2 (class-II, which do not have regulatory subunits), by contrast, significantly reduced the frequency of fast-moving anterograde runs (Fig. S6, Table S1; Kolmogorov–Smirnov test, *P*<0.001), although there was a significant increase in the anterogradely moving fraction of Rab4 vesicles in the axons (Fig. S6C). Finally, blocking PI3K-mediated signaling with all inhibitors and RNAi lines – except PI3KC2 – resulted in elevated Rab4 vesicle density in distal cholinergic axons (Figs S5B, S6B), corroborating the effect observed with dInR loss (Fig. S3G).

In summary, the results indicate that each PI3K subtype influences Rab4 vesicle movements in axons in a specific manner and Vps34 appears to have a direct role in accelerating the anterograde movement of a subset of these vesicles downstream of insulin signaling. These findings provided a new perspective for the mechanism underlying the insulin-dependent regulation of Rab4 vesicle movements in axons.

### Vps34-dependent and PI(3)P-mediated signaling can regulate the velocity of Rab4 vesicles in axons

Vps34 is an early endosome-localized (Sato et al., 2001) lipid kinase that specifically produces PI(3)P from phosphatidylinositol both *in vitro* (Bago et al., 2014) and *in vivo* (Schu et al., 1993). Furthermore, cellular PI(3)P can be readily detected by a genetically encoded 2×FYVE–GFP biosensor (Pattni et al., 2001). After showing that insulin-dependent Vps34 activation promotes Rab4 vesicle transport, we next tested whether insulin could activate Vps34 in cholinergic neurons. For this purpose, we used tissue-specific expression of 2×FYVE–GFP, a PI(3)P biosensor, as a proxy – allowing us to distinguish it from the standard insulin biosensor that reports activation of class-I PI3K, Akt or other downstream effectors (Britton et al., 2002; Shimizu-Sato et al., 2002; Ting et al., 2001; Wang et al., 2005; Gao and Zhang, 2008).

**Fig. 6.**
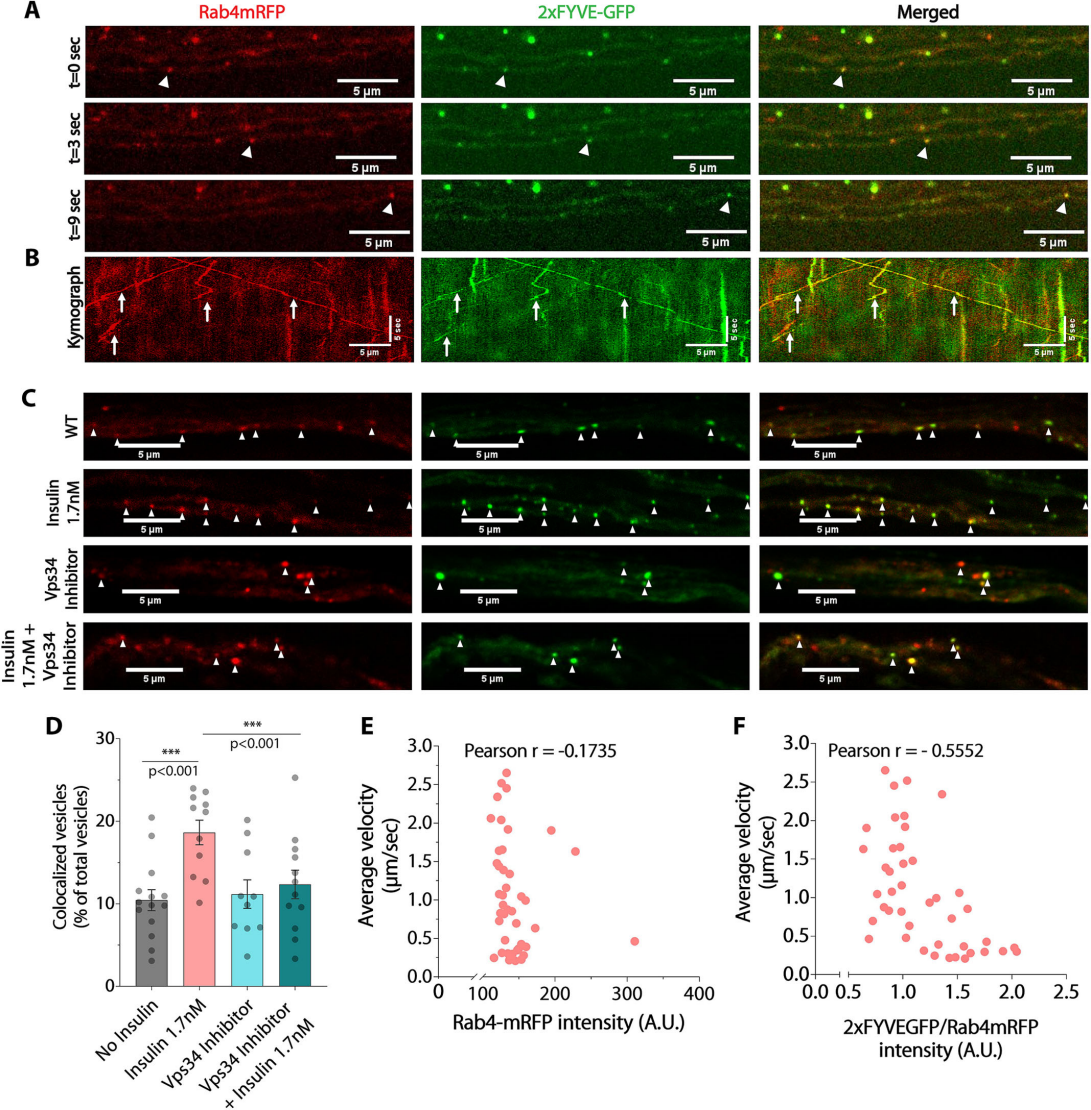
**Vps34-mediated endosomal PI(3)P-signaling regulates axonal transport of Rab4 vesicles downstream of insulin signaling.** (A,B) Simultaneously acquired dual-channel time-lapse images of segmental nerves (A) depicting migration of a vesicle [arrowhead marked with Rab4–mRFP (red) and 2×FYVE–GFP (green, PI(3)P biosensor)] and kymographs with arrows highlighting several colocalized tracks (B) in cholinergic axons. (C) Optical slices of segmental nerves of wild-type control with no insulin (WT) and after the insulin and class-III PI3K inhibitor (SAR405) treatments depicting colocalization (arrowheads) of Rab4–mRFP (red) and 2×FYVE–GFP (green) on vesicles in axons. (D) Percentage of colocalized vesicles (mean±s.e.m.) of the total (Rab4–mRFP and 2×FYVE–GFP) in wild-type control and different treatment backgrounds (*n*≥11 ROIs, *N*=3 larvae, >1000 vesicles each). The pairwise significance of difference was estimated using the Mann–Whitney *U*-test. (E,F) Correlations between Rab4–mRFP intensity and corresponding average velocities obtained using single-particle tracking (E), and between the 2×FYVE–GFP/Rab4–mRFP intensity ratios and corresponding average velocities (F); *n*=43 vesicles, *N*=6 larvae of Rab4 vesicles. A.U., arbitrary units.

Dual-color time-lapse imaging of cholinergic neurons co-expressing Rab4–mRFP and 2×FYVE–GFP revealed numerous motile axonal vesicles carrying both markers (Fig. 6A,B; Movie 6). Using fixed larval preparations, we further identified that nearly 10% of the combined pool of 2×FYVE–GFP and Rab4 vesicles (see Materials and Methods for details) were dually marked with Rab4–mRFP and 2×FYVE–GFP in the control preparations (Fig. 6C,D).

Next, we assessed that acute insulin stimulation significantly increased the fraction of the colocalized vesicles (Fig. 6C,D), which was also visually confirmed using anti-Rab4 antibody staining in the *cha>2xFYVEGFP* background (Fig. S7A). As expected, acute treatment with Vps34-specific SAR405 inhibitor abrogated the insulin-dependent increase in the percentage of colocalized vesicles (Fig. 6C,D). A similar acute treatment with Vps34 inhibitor also reduced the number of PI(3)P puncta in the larval VNC (Fig. S7B). These findings suggest that Vps34 activation downstream of neuronal insulin signaling can induce PI(3)P formation on Rab4 vesicles and confirm that the proposed use of the proxy reporter could be utilized to study the activation of the Insulin-Vps4-PI(3)P axis *in situ*. The formation of PI(3)P on the endosome surface activates PI(3)P-mediated lipid signaling (Tsukazaki et al., 1998). Hence, the data indicated the role of Vps34 on accelerating Rab4 vesicle movement downstream of InR signaling (Table S1) and further suggested that increased PI(3)P on the Rab4 vesicles could enhance their anterograde velocity and run length.

To further test this conjecture, we harnessed the previously documented ability of 2×FYVE–GFP to competitively mask the binding of endogenous PI(3)P-binding proteins (Gillooly, 2000). Further quantification of Rab4-mRFP intensity on individual motile vesicles revealed a consistent distribution in both control (*cha*>*Rab4mRFP*) and co-expression (*cha*>*Rab4mRFP*, *2xFYVEGFP*) backgrounds (Fig. 6E; Fig. S7C). Notably, Rab4–mRFP levels (a proxy for Rab4 activation) showed no correlation with vesicle velocity in either direction, consistent with evidence that Rab4 activity recruits both kinesin-2 and dynein motors (Imamura et al., 2003; Bielli et al., 2001). This observation implies that additional rate-limiting factors might regulate Rab4 vesicle movement in axons. In contrast, vesicle velocity decreased proportionally with increasing 2×FYVE–GFP localization (Fig. 6F), indicating a negative correlation between PI(3)P biosensor intensity relative to Rab4 and vesicle speed (Movie 7). Together with the effect of Vps34 knockdown on anterograde Rab4 transport, these findings indicate that PI(3)P biosensor binding might displace endogenous PI(3)P-binding proteins required for sustaining anterograde motility.

Altogether, the data suggests that the recruitment of PI(3)P-dependent factors on the surface of Rab4 vesicles could likely enhance the anterograde velocity of Rab4 vesicles downstream of insulin receptor signaling. These interpretations are also consistent with the reported suppression of anterograde-specific movement of the Rab4 vesicles upon Vps34 inhibitor treatment and in the Vps34 RNAi background (Fig. 5; Fig. S5). Hence, we conjecture that insulin signaling might promote the recruitment of a plus-end-directed kinesin motor on Rab4 vesicles through PI(3)P. Furthermore, these results also illustrated that increased localization of PI(3)P biosensor, 2×FYVE–GFP, on Rab4 vesicles could be a credible indicator of the insulin-dependent activation of InR-Vps34-PI(3)P pathway in cholinergic axons of *Drosophila* larvae.

### Loss of Kinesin-2 function fails to block the insulin-stimulated increase in the anterograde transport of Rab4 vesicles

To identify the kinesin motor that could likely play a role in regulating axonal transport of Rab4 vesicles downstream of insulin signaling in the axons, we chose to investigate the role of heterotrimeric Kinesin-2 as the first candidate. Previous reports have established this motor as a key regulator of Rab4 vesicle transport in cholinergic neurons (Dey et al., 2017) and of the GLUT4 vesicles in adipocytes (Imamura et al., 2003). Using dual-channel live imaging of cholinergic neurons expressing Rab4–mRFP and Klp64D–GFP (the KIF3A ortholog in *Drosophila* and an essential subunit of Kinesin-2), we demonstrate that Kinesin-2 likely comigrates with Rab4 vesicles in the axons (Fig. S8A,B). However, these events were infrequently detected due to very high cytoplasmic backgrounds of Klp64D–GFP (Fig. S8A). We then used a previously characterized *Klp64D* mutant (*Klp64D^K5^*), known to affect Kinesin-2 function *in vivo* (Ray et al., 1999), to perturb the transport of Rab4 vesicles in the axons. Consistent with the previous report on lch5 neurons of *Drosophila*, which showed a significant decrease in the density, anterograde fraction, and segmental velocity of Rab4 vesicles in the *Klp64D^K5^* homozygous background (Dey et al., 2017), the density of Rab4 vesicles reduced significantly and the anterograde movement of Rab4 vesicles was moderately affected in the cholinergic neurons in the heterozygous (*Klp64D^K5^/+*) background (Fig. S8C–I). In addition, we noted a significant increase in the pool of stationary vesicles (Fig. S8E), suggesting a likely role of Kinesin-2 in the initiation of Rab4 vesicle transport. As expected, we also observed a significant reduction in the run length (Fig. S8F,G) and the frequency of fast-moving runs of Rab4 vesicles both in the anterograde and retrograde directions (Fig. S8F,G, Table S1; Kolmogorov–Smirnov test, *P*<0.001).

Together, these observations suggested that *Klp64D^K5^* mutation could act as a dominant-negative allele and suppress the Kinesin-2 function in transport of Rab4 vesicles. However, contrary to the expectations, acute insulin stimulation in the *Klp64D^K5^/+* background rescued the motility of the stationary pool and elevated the anterograde fraction of Rab4 vesicles to the expected level (Fig. S8E). Such a rescue would ideally not be possible if Kinesin-2 was the motor responsible for insulin-mediated increase in anterograde fraction of Rab4 vesicles. Hence, these results suggest that although Kinesin-2 could play a critical role in promoting the Rab4 vesicle transport, a different anterograde motor is likely involved in insulin-stimulated acceleration of a subset of Rab4 vesicles in the axons.

### Klp98A recruitment via PI(3)P-signaling activates anterograde transport of Rab4 vesicles downstream of insulin stimulation

**Fig. 7.**
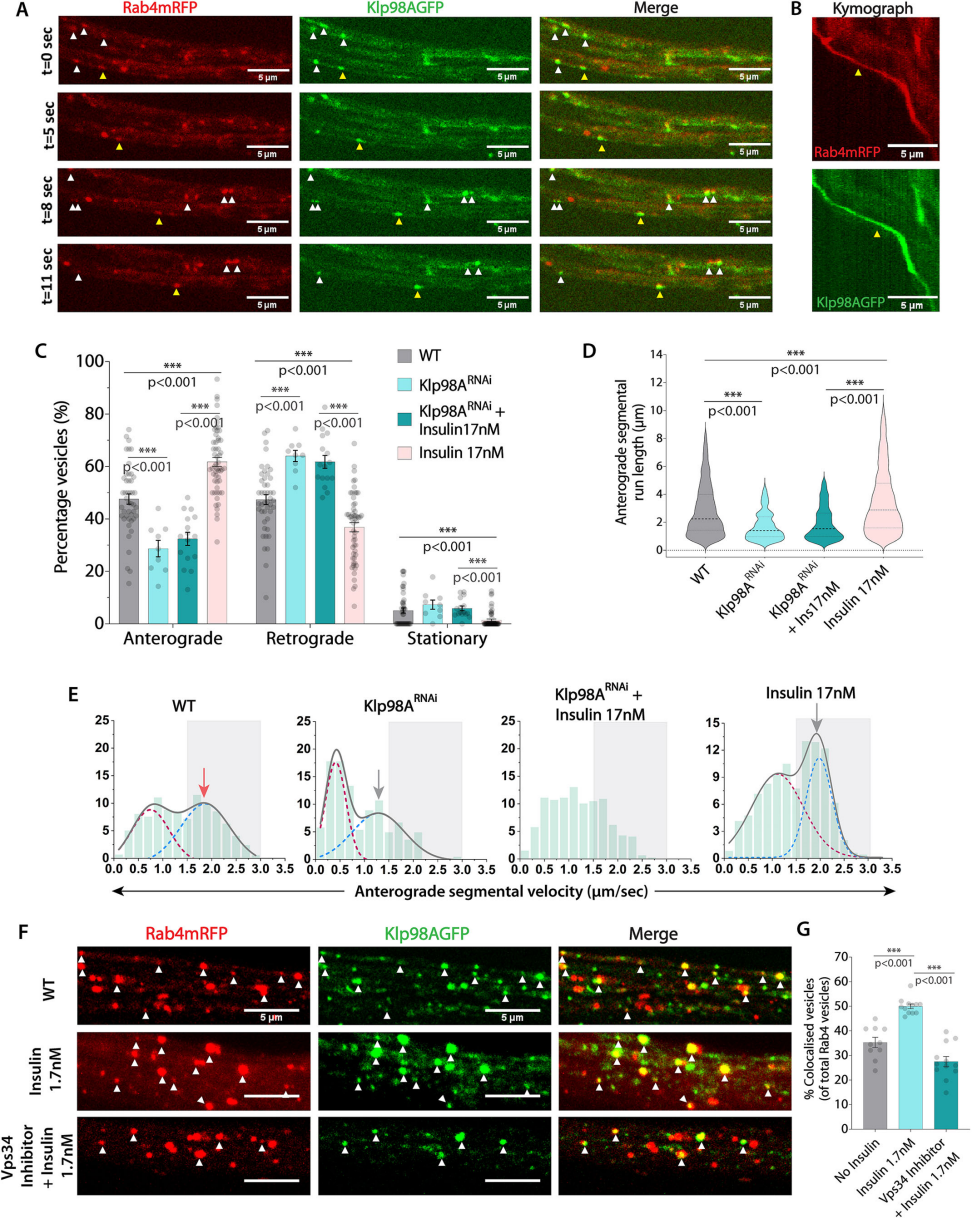
**The KIF16B ortholog Klp98A is recruited through a Vps34 and PI(3)P-dependent pathway downstream of insulin signaling and increases the anterograde fraction of Rab4 vesicles.** (A,B) Dual-channel time-lapse images of segmental nerve (A) depicting migration of vesicles marked by Rab4–mRFP and Klp98A–GFP (white and yellow arrowheads) where yellow marks the vesicle highlighted in the kymograph (B) in a cholinergic axon. (C–E) Relative distributions of the Rab4 vesicle movement (C, *n*≥9 segmental nerves, *N*=4–5 larvae), anterograde segmental run lengths (D) and anterograde segmental velocity distributions (E, *n*>200 runs) in the wild-type control (WT) and Klp98A^RNAi^ backgrounds in the absence and presence of insulin at 90 h AEL. C is shown as mean±s.e.m., and D as violin plots with dashed lines highlighting median and quartiles. The cumulative velocity distributions in E (gray) are shown as a sum of two Gaussians highlights slow (maroon) and fast-moving (blue) populations. Gray arrows highlight peak values of fast-moving populations of treatment groups; red arrow highlights peak values of fast-moving populations of the control group. (F) Optical slices of segmental nerve axons in control (WT), and after acute treatment with insulin, and insulin plus Vps34 inhibitor depicting the colocalization of Rab4–mRFP and Klp98A–GFP. (G) Fraction (mean+s.e.m.) of vesicles with colocalization of Rab4–mRFP and Klp98A–GFP in different treatment backgrounds (*n*≥10 segmental nerves, *N*≥3 larvae, ≥1800 vesicles). The pairwise significance of difference was estimated using a two-tailed unpaired *t*-test.

Next, we focused on Klp98A, a KIF16B ortholog and the only kinesin with a PI(3)P-binding (PX) domain in the C-terminal ‘tail’ region (Hoepfner et al., 2005). First, we demonstrated that ectopically expressed Klp98A–GFP marked and comigrated with several Rab4–mRFP vesicles in the axons (Fig. 7A; Movie 8). Next, cell-specific Klp98A knockdown significantly reduced the anterograde fraction, run length, and segmental velocity of Rab4 vesicles (Fig. 7C–E; Fig. S9A, Table S1). As observed previously, it also led to a significant reduction of retrograde segmental run length and a moderate reduction in retrograde velocity (Fig. S9C,D), which was much less severe than the loss of anterograde transport parameters. Finally, acute insulin stimulation could not increase the anterograde fraction, run length, or velocity of Rab4 vesicles in the Klp98A^RNAi^ background (Fig. 7C–E). Altogether, these observations suggested that insulin stimulation could help recruit Klp98A onto the Rab4 vesicles through Vps34/PI(3)P signaling to accelerate the anterograde transport of Rab4 vesicles.

To test this hypothesis, we estimated the number of Rab4–mRFP vesicles labelled with Klp98A–GFP in segmental nerve axons in the presence and absence of insulin. It revealed that acute insulin stimulation could significantly increase the frequency of Klp98A–GFP localization on Rab4 vesicles, which was completely abrogated in the presence of Vps34-specific inhibitor (Fig. 7F,G; *n*≥10 segmental nerves, *N*≥3 larvae, >1800 total vesicles). Molecularly, our data demonstrated that Klp98A recruitment on Rab4 vesicles could be regulated by the insulin-dependent activation of Vps34 (Fig. 7H), which would be a crucial step in increasing the anterograde velocity, processivity and fraction of Rab4 vesicles in the axons. These results also implicated Klp98A (KIF16B) in accelerating the Rab4 vesicles towards synapse upon insulin stimulation. Additionally, our results also suggested that Kinesin-2 could play an important role in initiating anterograde movement of Rab4 vesicles (Fig. S8C). In effect, a concerted action of these two kinesin motors in the axon could propel the Rab4 vesicles towards the synapse. Finally, given the previous reports (Dey et al., 2017; Imamura et al., 2003), our data also imply that Rab4 activity has a crucial role of in recruiting both these kinesin motors on endosomal vesicles.

### PI(3)P-signaling and Klp98A recruitment on Rab4 vesicles is developmentally regulated during the synaptic remodeling phase in VNC

**Fig. 8.**
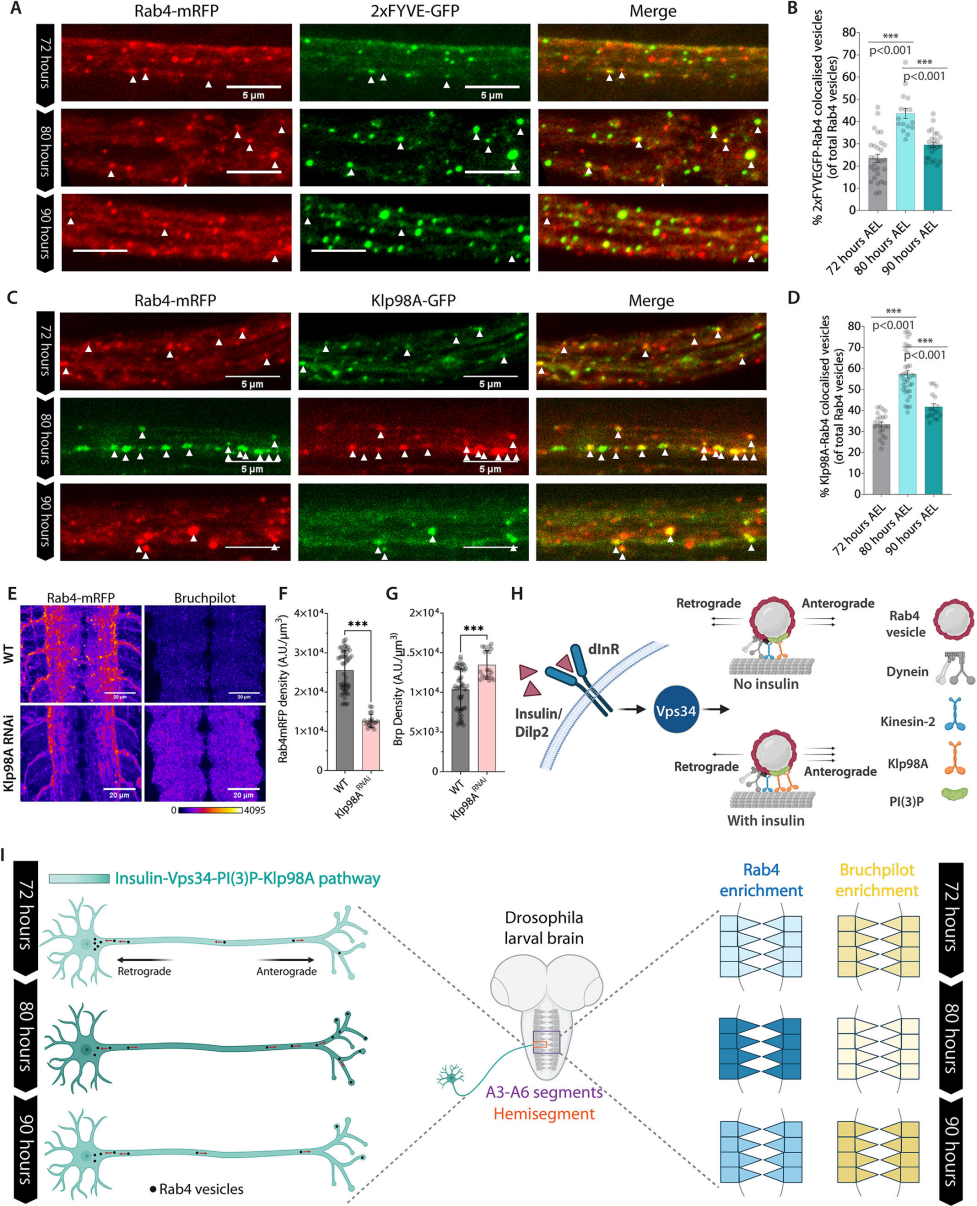
**Klp98A and PI(3)P levels on Rab4 vesicles are developmentally regulated with the same timing.** (A) Optical slices of segmental nerve axons depicting colocalization between Rab4 (red) and 2×FYVE–GFP (green) on axonal vesicles (arrowheads) during 72–90 h AEL. (B) Percentage (mean±s.e.m.) of Rab4 vesicles colocalized with PI(3)P biosensor at 72–90 h AEL (*n*>16 segmental nerves, *N*=3–6 larvae). The pairwise significance of the difference is estimated using a two-tailed unpaired *t*-test. (C) Optical slices of segmental nerve axons depicting colocalization between Rab4 vesicles (red) and Klp98A–GFP (green) during 72-90 h AEL. (D) Percentage (mean±s.e.m.) of Rab4 vesicles colocalized with Klp98A–GFP during 72–90 h AEL (*n*>17 segmental nerves, *N*=3-6 larvae). The pairwise significance of the difference is estimated using a two-tailed unpaired *t*-test. (E–G) The effects of Klp98A RNAi in cholinergic neurons on Brp and *cha>Rab4mRFP* localizations in the VNC at 80 h AEL. Note that the Klp98A RNAi induced a significant loss of Rab4mRFP levels in the neuropil regions contributed by the cholinergic neurons and an increase in the global Brp levels in the neuropil. The relative loss of Brp was lesser because the RNAi was effective only in the cholinergic neurons which contributes to nearly 50% of the total synapses in the VNC. Results are mean±s.d. (*n*>20). *P*<0.001 (Mann–Whitney U test). A.U., arbitrary units. (H) The model illustrates the molecular effects of insulin signaling on a subset of Rab4 vesicles in cholinergic axons. Created in BioRender by Singh, K., 2025. https://BioRender.com/p1c28o1. This figure was sublicensed under CC-BY 4.0 terms. (G) The schematic summarizes the observations described so far and suggest that developmental alterations of insulin signaling could regulate the anterograde flow of a subset of Rab4 vesicles involved in synaptic remodeling. Created in BioRender by Singh, K., 2025. https://BioRender.com/hzw1rg9. This figure was sublicensed under CC-BY 4.0 terms.

The results described so far clearly establish that the InR-Vps34-Klp98A axis could accelerate the synapse-bound movement of a subset of Rab4 vesicles marked with PI(3)P. Also, we showed that PI(3)P and Klp98A–GFP levels increase on the Rab4 vesicles in axons due to the dInR activation. Hence, to understand whether this pathway could indeed account for the programmed changes in the Rab4 vesicle movement during 72-90 h AEL as reported at the beginning of this article, we studied the developmental changes in the 2xFYVEGFP and Klp98AGFP levels on Rab4 vesicles in axons using fixed tissue preparations of transgenic larvae at 72, 80, and 90 h AEL. First, we observed a significant increase in the normalized fraction of vesicles with colocalized Rab4–mRFP and 2×FYVE–GFP, relative to the total number of Rab4 vesicles, from 72–80 h AEL, which subsided at 90 h AEL (Fig. 8A,B), indicating a developmentally regulated increase of the Vps34 activation in cholinergic neurons at 80 h AEL. A similar episodic increase in the normalized fraction of Rab4–mRFP and Klp98A–GFP colocalized vesicles, relative to the total number of Rab4–mRFP vesicles, was observed from 72–80 h AEL, which subsided at 90 h AEL (Fig. 8C,D), which coincided with the observed increment of anterograde flux of Rab4 vesicles during 80 h AEL (Fig. 2D). Taken together, these results indicate that episodic increase of Vps34-dependent Klp98A recruitment of Rab4 vesicles in cholinergic neurons could regulate the periodic Rab4 enrichments in the VNC during the episodic reduction of gross synaptic density.

To test the loss of InR-Vps34-Klp98A pathway activity in cholinergic neurons on synaptic density in the larval CNS, we decided to knockdown Klp98A. This was specifically done to minimize expected off-target effects upon perturbation of InR or Vps34 as they have independently been shown to regulate synaptic homeostasis via a wide range of downstream effectors and pathways (Lee et al., 2011; Sánchez-Castillo et al., 2022; Hu et al., 2023; Karpova et al., 2025). Therefore, we estimated the effect of the Klp98A RNAi in cholinergic neurons on Rab4 enrichment and synapse density in the VNC neuropil at 80 h AEL. As expected, it revealed a significant reduction in Rab4–mRFP levels (Fig. 8E,F; Fig. S9E) and an increase in synaptic density (Fig. 8E–G; Fig. S9E). The Rab4–mRFP puncta in the synaptic region of the neuropil were visibly reduced and those marked by Brp were more numerous (Fig. S9E). Together, these results suggest a causal link between Vps34-driven changes in Klp98A recruitment in cholinergic neurons and the localization of motor on a subset of Rab4 vesicles. This recruitment leads to their enrichment at presynaptic terminals and is closely associated with episodic reductions in synaptic density within the VNC (Fig. 8H,I). Also, a concerted increase of both the biomarkers (i.e. Vps34 and Klp98A), established to act downstream of dInR signaling in cholinergic neurons further helped to conjecture a potential role of episodic increase of insulin signaling in these neurons.

## DISCUSSION

### Effects of neuronal insulin signaling on Rab4-associated recycling endosomes in axons

Endosomal trafficking and recycling in neurons are essential for neuronal development and survival (Schmidt and Haucke, 2007; Yap and Winckler, 2012). The directed movement of endosomal traffic is a crucial regulator of several neuronal processes like signaling, autophagy, synaptic vesicle recycling and neurotransmission (Liu et al., 2022; Olenick et al., 2019; Zhao and Zhang, 2019). Besides, perturbations in endosomal functions are one of the earliest pathologies in neurodegenerative disorders like Alzheimer's disease (Nixon, 2005). However, signaling cues regulating directed endosomal movement in the axons remain relatively understudied. In this context, we show that developmental regulation of neuronal insulin signaling could regulate the directionality and overall flow of a subset of endosomal vesicles in axons.

Insulin signaling in *Drosophila* brain has been implicated in age-related cognitive decline (Augustin et al., 2017). Furthermore, insulin signaling in neurons has been shown to regulate synaptic density (Chiu et al., 2008), neurodegeneration (Schubert et al., 2004), aging (Kenyon, 2010) and behavior (Kleinridders and Pothos, 2019) in various other model systems. However, its effect on long-range axonal transport, which is essential for maintenance of synaptic homeostasis, remained unknown. Using ectopic stimulation and tissue-specific perturbations, we show that signaling via insulin, Dilp2 and dInR could accelerate the anterograde movement of a subset of Rab4 vesicles in the cholinergic neurons of *Drosophila*. This observation could have substantial physiological implications, as increased accumulation of activated Rab4 at the presynaptic terminals is known to adversely affect synaptic density in *Drosophila* CNS (Dey et al., 2017), and elevated Rab4 levels in both CBF and hippocampal CA1 neurons are positively correlated with neurodegeneration and cognitive decline in Alzheimer's disease (Ginsberg et al., 2011, 2010). Our results, together with other findings, suggest that insulin signaling could manifest its effect on synapse organization, neurodegeneration and aging in part via altered trafficking of Rab4 endosomes.

The experimental data also suggest that insulin-dependent redirection of Rab4 might regulate neuropil growth and synaptic remodeling in the larval VNC. Rab4 plays an integral role in neurite outgrowth, recycling of surface receptors, and regulating endosomal traffic inside a cell (van der Sluijs et al., 1992; Falk et al., 2014; Hoogenraad et al., 2010; De Wit et al., 2001). Thus, increased enrichment of Rab4 at the presynaptic terminals could alter recycling of synaptic vesicles and promote neurite outgrowth, consequently regulating synaptic strength and stability. Such a process might also modify the composition of presynaptic membrane and alter neurotransmission. Here, utilizing different GAL4 drivers or generating single neuron clones that help in analyzing the effect of axonal transport on synapses at a higher resolution in the future might help in uncovering the cellular mechanisms that could explain how increased Rab4 accumulation at the presynaptic terminals affect synaptic stability.

### Insulin signaling preferentially activates Vps34, increasing PI(3)P on a subset of Rab4 vesicles in axons

This study also identified a selective activation of Vps34 downstream of insulin signaling in the axons. Insulin signaling is known to activate PI3Ks, particularly the class I PI3K in peripheral tissues (Boucher et al., 2014), which activates Rab4 and early endosomal recycling (Chamberlain et al., 2010). Here, we show that preferential activation of the class III PI3K Vps34 by insulin in axons accelerates movement of a subset of Rab4 vesicles towards the synapse. Thus, the mechanism activating the Rab4 vesicle trafficking downstream of the insulin receptor appears to be quite distinct in the axons as compared to that observed in adipocytes.

In addition, throughout our study, we also noted that the effect of InR-Vps34-PI(3)P pathway is observed only on ∼15–20% of the total Rab4 vesicles in the axons. This is interesting, especially in light of a recent study from the motor neurons of *Drosophila*, which has shown that there is content heterogeneity of the Rab4 vesicles with only a small proportion carrying synaptic and axonal cargoes (White et al., 2020). Hence, one would expect various cell signaling pathways to only mobilize selective fractions of Rab4-associated endosomes carrying specific types of cargo in the axon. Therefore, it would be interesting to further probe the mechanism of Rab4 vesicle targeting in axon by the InR-Vps34 signaling.

Vps34 – the lipid kinase that exclusively produces PI(3)P inside the cell – plays a key role in regulating neuronal processes like autophagy (Juhász et al., 2008), synaptic vesicle recycling (Cremona et al., 1999), neurotransmission (Li et al., 2019) and neuronal survival (McKnight et al., 2014). Recent studies have also shown that PI(3)P-positive endosomes have a crucial role in regulating synaptic vesicle recycling and neurotransmission (Liu et al., 2022). Further, a nutrient-sensitive, PI(3)P-mediated lipid signaling on endosomes has been suggested to regulate endoplasmic reticulum (ER) shape and mitochondrial function (Jang et al., 2022). However, the source, identity and mechanism(s) regulating PI(3)P formation on endosomes in these studies was unclear. Our study has shown that neuronal insulin signaling promotes Vps34-dependent acceleration of Rab4-PI(3)P vesicles towards the synapse, which could also serve as an active source of replenishing the pool of PI(3)P-Rab4 dual-positive vesicles enriched at the presynaptic compartment.

### Klp98A recruitment and selective augmentation of synapse-directed Rab4 vesicle movement in axon

Previous work using a candidate-based screen for several Kinesin-1, Kinesin-2 and Kinesin-3 motors has shown that Kinesin-2 is the major regulator of Rab4-associated vesicular transport in the axons (Dey et al., 2017). However, the effect of the kinesin-3 motor Klp98A (the homolog of KIF16B), in regulating axonal transport of Rab4 vesicles remains unclear. Our findings suggest that a pool of Rab4-PI(3)P vesicles are accelerated through Klp98A recruitment on these vesicles. These motors contain a PI(3)P-binding PX-domain in the C-terminal tail (Ueno et al., 2011). Klp98A recruitment onto Rab4 vesicles in the axons due to Vps34 activation downstream of acute insulin stimulation increases their speed by nearly twofold. Consistent with this observation, an unbiased proteomic screen has also identified Vps34 as one of the Rab4 effectors (Gillingham et al., 2014).

A previous study in mouse hippocampal neurons has shown that KIF16B is required for somatodendritic localization of early endosomes, which helps in the trafficking of AMPA and NGF receptors (Farkhondeh et al., 2015). However, whether there is any role of KIF16B in regulating long-range axonal transport *in vivo* was unclear. We also noted that not all motile Klp98A-positive vesicles in axons were also positive for Rab4, indicating that Klp98A transports other cargoes apart from Rab4 vesicles in axons. Interestingly, mutations in the cargo-binding PX domain of KIF16B have recently been reported in individuals with intellectual disability syndrome (Alsahli et al., 2018), although the underlying cause remains to be investigated. Thus, our results could provide a crucial mechanistic input because perturbations in Rab4 levels and functionality also affect synaptic homeostasis, which happens to be one of the hallmarks of intellectual disabilities (Zoghbi and Bear, 2012). Therefore, it will also be worth investigating in the future if there is a synergistic interaction between Rab4 and Klp98A, keeping in mind their overlapping functions and phenotypes in the CNS.

Finally, cell biological studies have suggested that KIF16B drives the fission of early endosomes to form tubules (Skjeldal et al., 2012), receptor recycling and degradation (Hoepfner et al., 2005), transcytosis (Perez Bay et al., 2013) and polarized transport of growth factor receptors (Ueno et al., 2011). In addition, the Klp98A motor is implicated in the maturation of autophagic vesicles by promoting fusion through a motor-independent function (Mauvezin et al., 2016). The formation of PI(3)P on Rab11 vesicles upon starvation has been shown to serve as a platform for autophagosome formation in HeLa cells (Puri et al., 2018). However, such platforms and/or the identity of PI(3)P membranes required for autophagosome formation in neurons were not identified. In this context, it can be conjectured that the activation of Vps34-PI3P-Klp98A-dependent movement of a specific pool of Rab4 vesicles towards the synapse in *Drosophila*, as revealed by the above data, could serve as a platform for regulating autophagosome formation at the synapse and reduce the overall synaptic volume. This supposition is also consistent with the results indicating an inverse correlation between Klp98A-dependent Rab4 enrichment at the presynaptic terminals and synaptic density in a developing *Drosophila* larva.

### Axonal transport of a pool of Rab4 vesicles regulates synaptic remodeling in the CNS of *Drosophila* larvae

This study aimed to correlate the change in synaptic arborization in the CNS to the axonal transport of Rab4 vesicles in developing *Drosophila* larvae. The phenomenon observed provides a statistically significant correlation between the observed changes in the Brp (the homolog of ELKS) levels and Rab4–mRFP enrichments in the VNC neuropil to that of the synapse-directed movement of a subset of Rab4-associated vesicles in axons. The study also showed a clear correlation between the changes in PI(3)P and Klp98A localization on these vesicles during the same period. Altogether the data suggests that a developmentally regulated increase in Rab4 vesicle movement towards the synapse coincides with the loss of Brp enrichment at the neuropil. We have interpret these data as showing that increased Rab4-associated endosomal transport potentially abrogates presynaptic arborization and resorbs the synapses during development. The phenomenon is also observed in a developing system, which highlights its biological significance in a physiologically relevant context. In our previous study, we showed that reduced Brp staining in the Rab4^CA^ overexpression background correlates with an increased persistence length of the larval locomotion (Dey et al., 2017). It is also important to note that third-instar larvae are highly motile at the mid-late stages, when they frequently skim food surface and dive back before exiting the food for pupariation. However, a systematic study is needed to correlate this behavior with the VNC data.

## MATERIALS AND METHODS

### *Drosophila* stocks and rearing

All fly stocks were obtained from Bloomington *Drosophila* stock center unless mentioned otherwise and were reared at 25°C on standard corn agar meal with a 12-h-light–12-h-dark cycle. Eggs were collected for 1 h and kept at 25°C for 72, 80 or 90 h. Fly stocks used were: Brp-GFP (BL59292), *UAS Rab4mRFP* (BL8505), *UAS dsRNA InR* (BL31594, Valium1), *UAS dsRNA InR* (BL51518, Valium20), *UAS InR^CA^* (BL8250), *UAS dsRNA PI3K92E* (BL27690), *UAS dsRNA PI3K21B* (BL38991), *UAS dsRNA PI3K68D* (BL35265), *UAS dsRNA PI3K59F* (BL33384), *UAS GFP-myc-2xFYVE* (BL42712), *Klp64D^K5^* (Ray et al., 1999) and *UAS Klp64D-GFP* (Jana et al., 2011), *UAS dsRNA Klp98A* (BL 50542) and *UAS Klp98A-GFP* (Derivery et al., 2015).

### Larval fillet preparation – time-lapse imaging and fixed tissue preparations

Larvae were dissected in 1× HL3.1 buffer (Feng et al., 2004; containing 70 mM NaCl, 5 mM KCl, 1.5 mM CaCl_2_, 4 mM MgCl_2_, 10 mM NaHCO_3_, 5 mM Trehalose, 115 mM sucrose and 5 mM HEPES) and insulin (Sigma-Aldrich), Dilps (Pheonix Pharmaceuticals Inc.), PI3K inhibitors (Sigma-Aldrich) (see below for details) were finally reconstituted in HL3.1 buffer for treatment followed by mounting on the coverslip cavity chamber. A small piece of tissue drenched in water was placed on the side of the Petri dish to maintain humidity. The dish was then covered and sealed with Parafilm and immediately taken for time-lapse imaging. Epifluorescence and spinning disc confocal (SDC) time-lapse imaging was performed using Nikon Ti Eclipse TIRF Microscope (installed with a Yokogawa CSU-W1 disk with pinhole size 50 µm for SDC microscopy) operated with running Nikon Elements software using a 100× (1.4 NA) oil-immersion objective (binning=1) with Andor iXon EMCCD camera and sCMOS camera (Zyla 4.2 plus sCMOS) respectively.

Live movies were recorded for all conditions at a frame rate of 8–10 frames per second (fps). Sample sizes for all our experiments throughout the study were collected from at least from three animals and at least ten different segmental nerve axons. As a general rule, live movies were recorded from 15 or more segmental nerves from five or more animals across all tested conditions (unless specified otherwise). However, given the intricate dissection procedure involved and the peristaltic movement of larval cuticle, which leads to drifts in the axons in many recorded movies, only a subset of the total data collected is analyzed and included in the study. For fixed tissue analysis, fillet preparations were immediately fixed in 4% paraformaldehyde (PFA) in 1× PBS for 15–20 min at room temperature (RT). Following fixation, they were rinsed three times with 1× PBS and mounted on a coverslip with a drop of VECTASHIELD antifade mounting medium (Vector Laboratories).

### Insulin, Dilp and drug treatments

Stock solutions of Insulin (Sigma, I0516) and Dilp2 or Dilp5 (Pheonix Pharmaceuticals Inc., 036-17 and 035-96) were diluted and prepared in HL3.1 buffer, respectively. Likewise, stock solutions of the non-specific PI3K inhibitor (5 mM) LY294002 (Abcam, #ab120243), HS173 (50 µM) (Sigma Aldrich, 5.32384) and SAR405 (5 mM) (Sigma-Aldrich, 5.33063) were prepared in DMSO (Sigma) and used at effective concentrations of 50 µM, 50 nM and 25 µM reconstituted in HL3.1 buffer, respectively.

### Quantification of axonal transport and statistical analyses

All live-imaging movies were analyzed on ImageJ/Fiji (https://imagej.net/Fiji). Segments were classified and parameters like fraction of cargo population, velocity, and run length were calculated using the KymoAnalyzer plugin for Fiji, as discussed in Neumann et al. (2017). Briefly, kymographs were manually traced to identify segments as a portion of a track for which the slope of the traces on kymographs remained constant before it stopped or changed direction. KymoAnalyzer then extracts the coordinates of those segments and tracks and calculates different parameters like distance and velocity using standard formulas to compute segmental run length and velocity. Fraction cargo population is calculated by first computing the net displacement of all vesicles from their start position and then classifying them into anterograde, retrograde or stationary based on their net displacement. Input pixel size and frame rate were used as per the movies recorded.

### Immunostaining

Dissected VNC of larvae were immediately fixed in 4% PFA in 1× PBS for 20 min at RT followed by three washes with 1× PBS, 10 min each. These samples were then permeabilized in 1× PBS containing 0.3% Triton-X-100 (PTX) for 20 min followed by blocking for an hour with 1 mg/ml bovine serum albumin (BSA) in 0.3% PTX (PBTX) at RT to block the non-specific reactive binding sites for antibody. Samples were then incubated with the primary antibodies diluted in PBTX for 2 h at RT followed by three 10-min washes in PBTX. Incubation with secondary antibodies diluted 1:400 in PBTX was undertaken for an 1 h followed by three 10-min washes in PBTX. Samples were finally mounted on a glass slide in a drop of Vectashield (Vector Laboratories Inc., USA) under an 18 mm×18 mm coverslip of 0.17 mm thickness. The same procedure was followed for immunostaining of fillet preparations. Antibodies, stains and dyes used were: anti:Brp (nc82, DSHB; 1:200), anti-GFP (ab290, Abcam; 1:1000), anti-Rab4 (ab78790, Abcam; 1:400), anti-mouse-IgG conjugated to Alexa Fluor 488 (A11029, A11008, Invitrogen; 1:400) and Hoechst 33342 (Sigma-Aldrich, 1:100).

### 3D rendering – volume and intensity estimation using Imaris software

All fluorescence images of Brp immunostained larval VNCs (except Fig. 8E) were collected under constant acquisition conditions using Zeiss LSM 510 Meta laser scanning confocal microscope, using a 40×1.3 NA objective at a pixel resolution of 0.62×0.62 μm^2^. Images for Fig. 8E for both wild-type and Klp98A RNAi were recorded under constant acquisition conditions using Olympus FV3000 using a 60×/1.42 NA objective with a pixel resolution of 0.2×0.2 μm^2^. Same secondary antibody was used across various time points and genotypes. The acquisition parameters viz., laser power, PMT gain, scan speed, optical zoom, offset, step size, pinhole diameter was kept constant for each experimental data set and samples (except for Fig. 8E) were processed in a single batch. Volume and total intensity measurements on 3D volume rendered image stacks were acquired using ImageJ (https://imagej.nih.gov/ij/) and Imaris software. A surface was reconstructed on the 3D volume rendered image using the surface module of the object menu of Imaris software for each neuromere hemisegment by manually selecting the regions. Intensity and volume measurements were taken from these surfaces.

### Electron microscopy

VNCs from larvae at 72, 80 and 90 h AEL were dissected in HL3.1 buffer and fixed overnight in 2.5% glutaraldehyde (EM Sciences), 4% paraformaldehyde, and 0.04% CaCl_2_ in 0.1 M phosphate buffer at 4°C. The tissues were washed in 0.1 M phosphate buffer and post-fixed in OsO_4_ for 4 h at 4°C, followed by washes in 0.1 M phosphate buffer (pH 7.4), dehydration in graded series of ethanol and embedding in Araldite (Merck). Ultrathin sections were obtained in Leica EM UC6, stained with aqueous uranyl acetate and lead citrate, and imaged using a Zeiss Libra 120 EF (Zoghbi and Bear, 2012).

### Colocalization estimation

Percentage of colocalized vesicles (Figs 6–8) were estimated using a standard automated ImageJ plugin ComDet v0.5.5 developed by Eugene Katrukha from Utrecht University (https://github.com/UU-cellbiology/ComDet). Imaging parameters for ComDet were chosen such that we could detect >95% vesicles in each image (adjusting pixel size, intensity threshold and distance between colocalized spots) and automated analysis was validated using manual counting for the control dataset.

### Quantitative PCR analysis to validate InR RNAi constructs

Wandering third-instar larvae were dissected to isolate larval brains (15 per condition) and collected in TRIzol buffer (Sigma-Aldrich) on ice. These larval brains were further processed using Qiagen RNeasy kit for RNA purification as per the manufacturer's protocol and eluted in nuclease-free water and stored in −20°C. The cDNA library was prepared from the isolated RNA using first-strand cDNA synthesis kit from Thermo Fisher Scientific as per manufacturer's protocol and quantitative PCR (qPCR) analysis was performed using KAPA Biosystems SYBR FAST Universal kit in Roche Light Cycler 480 II. qPCR experiments from 15 larvae per trial, repeated twice for all the genotypes (control, InR-RNAi-1 and InR-RNAi-2).

### Statistical information

Origin 2020 was used for plotting the frequency histograms (bin size 0.2 µm/s) and curve fitting was undertaken using multiple peak fit with the Gaussian peak function. GraphPad Prism 9.5.1 was used for plotting scatter plots and calculation of the Pearson coefficient (*r*). Nonparametric Kolmogorov–Smirnov tests were used for comparing all the frequency histograms for segmental velocities and were performed using Origin 2020. A Mann–Whitney *U*-test and unpaired two-tailed Student's *t*-test were performed using GraphPad Prism 9.5.1. The statistical tests used, ‘*N*’, and ‘*n*’ values are specified in the main text/figure legends for all the figures. Numerical raw data is shown in Tables S2–S11.

## Supplementary Material

10.1242/joces.264782_sup1 Supplementary information

TableS2. Raw data values for volume and intensity of Brp per hemisegment and segment-wise in A3-A6 from 72-90 hours AEL (Fig.1 D-E and fig. S1B-D) is organized in different sheets in the spreadsheet.

TableS3. Raw data values for volume and intensity of soluble GFP marked by *chaGal4* per hemisegment in A3-A6 (Fig.1 F-H) is organized in different sheets in the spreadsheet.

TableS4. Raw data values for density (intensity per unit volume) of Rab4 and Bruchpilot per hemisegment in A3-A6 (Fig. 2B).

TableS5. Raw data values of percentage of Rab4 vesicles in the anterograde, retrograde, and stationary categories (fraction) at different developmental timepoints, in different conditions, or after a pharmacological or genetic perturbation (Fig. 2E, 3A, 4A, 5A, 5D, 7C and fig. S4B, S6C, S8E).

TableS6. Raw data values of segmental run length (μm) of Rab4 vesicles in the anterograde and retrograde direction at different developmental timepoints, in different conditions, or after a pharmacological or genetic perturbation (Fig. 2F, S2A, 3B, 4B-C, 5B, E, 7D and fig. S3G, S4C-D, S5C, S6D-E, S8F-G, and S9C).

TableS7. Raw data values of segmental velocity (μm/sec) of Rab4 vesicles in the anterograde and retrograde direction at different developmental timepoints, in different conditions, or after a pharmacological or genetic perturbation (Fig. 2G, S2B, 3C, S3H, 4D, S4E, S5D, S5B, S6F, S8H-I, and S9D).

TableS8. Raw data values of anti-GFP intensity per unit area values (A.U. μm^-2^) in the segmental nerve axons for control and InR-CFP overexpression background (fig. S3C-D).

TableS9. Sheet 1: Raw data values of *percentage colocalized vesicles* of the total (Rab4mRFPand 2xFYVE-GFP) in different pharmacological treatments in wandering third instar larvae (Fig. 6C-D)and at different developmental timepoints (Fig. 8C-D). Sheet 2: Raw data values of *total Rab4vesicles* and *colocalized vesicle numbers* in different pharmacological treatments and at different developmental time points.

TableS10. Sheet 1: Raw data values of Rab4mRFP and 2xFYVEGFP intensities (A.U.) on single vesicles and their average velocities (μm/sec) in the distal axons of cholinergic neurons from the genotype *cha<Rab4mRFP, 2xFYVEGFP* (Fig. 6E-F). Sheet 2: Raw data values of Rab4mRFP intensities (A.U.) on single vesicles and their average velocities (μm/sec) in the distal neurons from the genotype *cha<Rab4mRFP/+* (Fig. S7C).

TableS11. Sheet 1: Raw data values of percentage *colocalized* vesicles of the total (Rab4mRFP and Klp98A-GFP) in different pharmacological treatments in wandering third instar larvae (Fig. 7F-G) and at different developmental timepoints (Fig. 8A-B). Sheet 2: Raw data values of total *Rab4* vesicles and *Rab4mRFP-Klp98AGFP* colocalized vesicle numbers in different pharmacological treatments and at different developmental time points.
