# Supplementary material for: Phosphatidylinositol-3-phosphate-dependent Klp98A recruitment regulates endosomal flux underlying developmental synaptic remodeling via Rab4
Source: J Cell Sci. 2026 Mar 27;139(6):jcs264782. doi: 10.1242/jcs.264782 (PMC13070254; doi:10.1242/jcs.264782)
Supplement: Supplementary information [file joces-139-264782-s1.pdf]

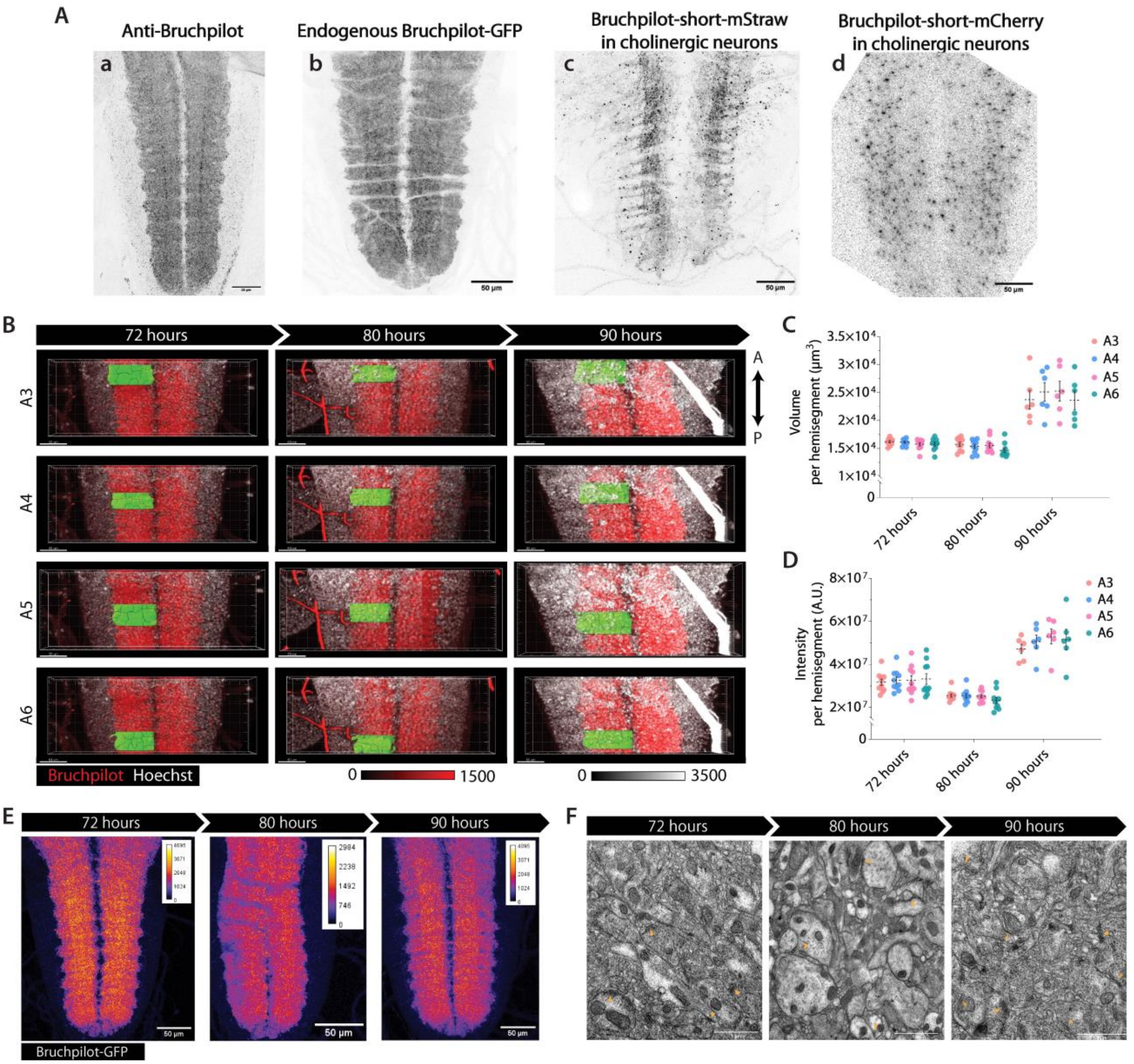

Fig. S1. The *Drosophila* central nervous system (CNS) undergoes synaptic remodeling during the 3rd Instar larva stage. (A) Synaptic regions of larval VNC marked with Ant-Brp immunostaining (a), endogenous Bruchpilot-GFP (MiMIC fly line 3BL59292) expression (b), and the expression of Brp-short-mStraw **(c)** and Brp-short-mCherry **(d)** by chaGal4. Note that the overexpression of Brp-short tagged with either mStraw or mCherry produced supernumerary puncta in cortex, neurites, and neuropil region. Also, the enrichment in the neuropil region was variable. Hence, they were ruled out for the estimation of gross synaptic content. **(B)** A3-A6 segments of larval VNC stained with anti-Bruchpilot (red) and Hoechst (white) at 72-90 hours AEL. **(C, D)** Mean

± SEM of synaptic volumes (C) and Bruchpilot enrichment (D) in A3-A6 hemisegments (N=3-5 larvae). **(E)** Electron micrographs of larval VNC at 72 (left), 80 (middle), and 90 hours (right) AEL where yellow arrowheads highlight active zones in synaptic boutons. Note the apparent increase in their number and electron density of active zones at 90 hours AEL (right), in agreement with Bruchpilot intensity and volume measurements.

Journal of Cell Science • Supplementary information

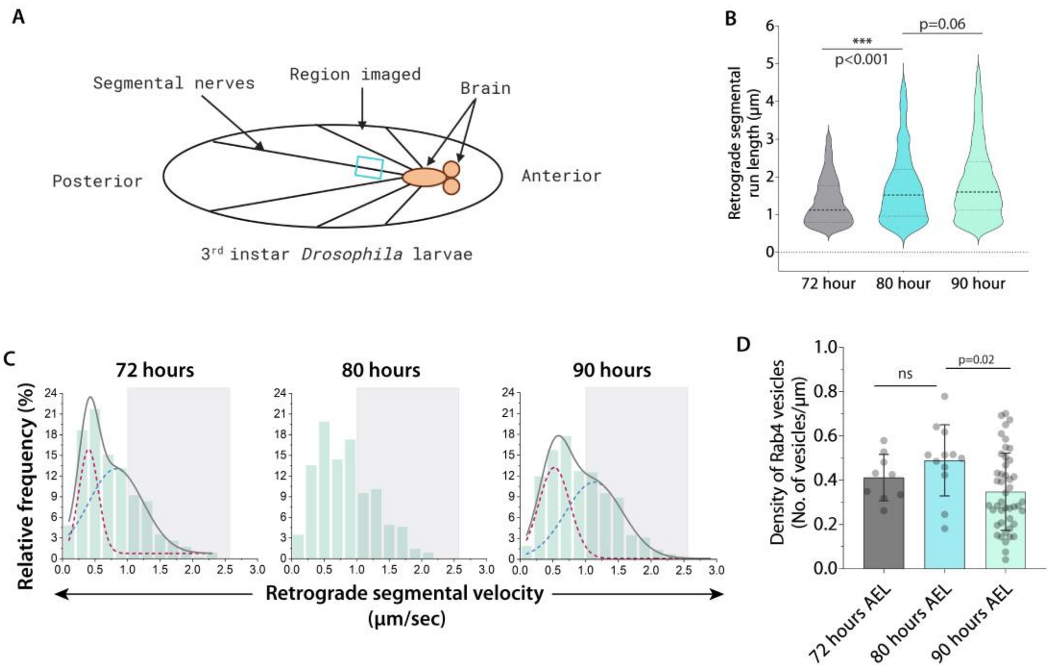

Fig. S2. Retrograde axonal transport of Rab4 vesicles in a developing larva from 72-90 hours AEL. (A) Schematic depicting the model system and the region of interest – distal axons of cholinergic segmental nerves of a third instar *Drosophila* larvae – from where the movies of axonal transport of Rab4 vesicles are recorded. Created in BioRender. Singh, K. (2025) https://BioRender.com/0dq6a69. **(B)** Retrograde segmental run length of Rab4 vesicles at 72-90 hours AEL (n>300 runs from N=3-5 larvae). **(C)** Retrograde segmental velocity distributions of Rab4 vesicles at 72-90 hours AEL (n>300 runs, N=3-5 larvae). The cumulative distribution (grey) as a sum of two Gaussians (maroon and blue dotted lines) highlights slow (maroon) and fast-moving (blue) populations (See methods for details). Grey box marks the fast-moving (≥ 1.5 μm/sec) runs. **(D)** Density of Rab4 vesicles (No. of vesicles/μm) in the distal axons of cholinergic neurons at 72, 80, and 90 hours AEL. Note: 90 hours AEL data column is the same as WT data (90 hours AEL) presented in Fig. S3I, Fig.S4F, Fig. S5B, Fig. S6B, Fig.S8D, and Fig. S9B. The pairwise significance of difference were estimated using the Mann-Whitney U-test.

Journal of Cell Science • Supplementary information

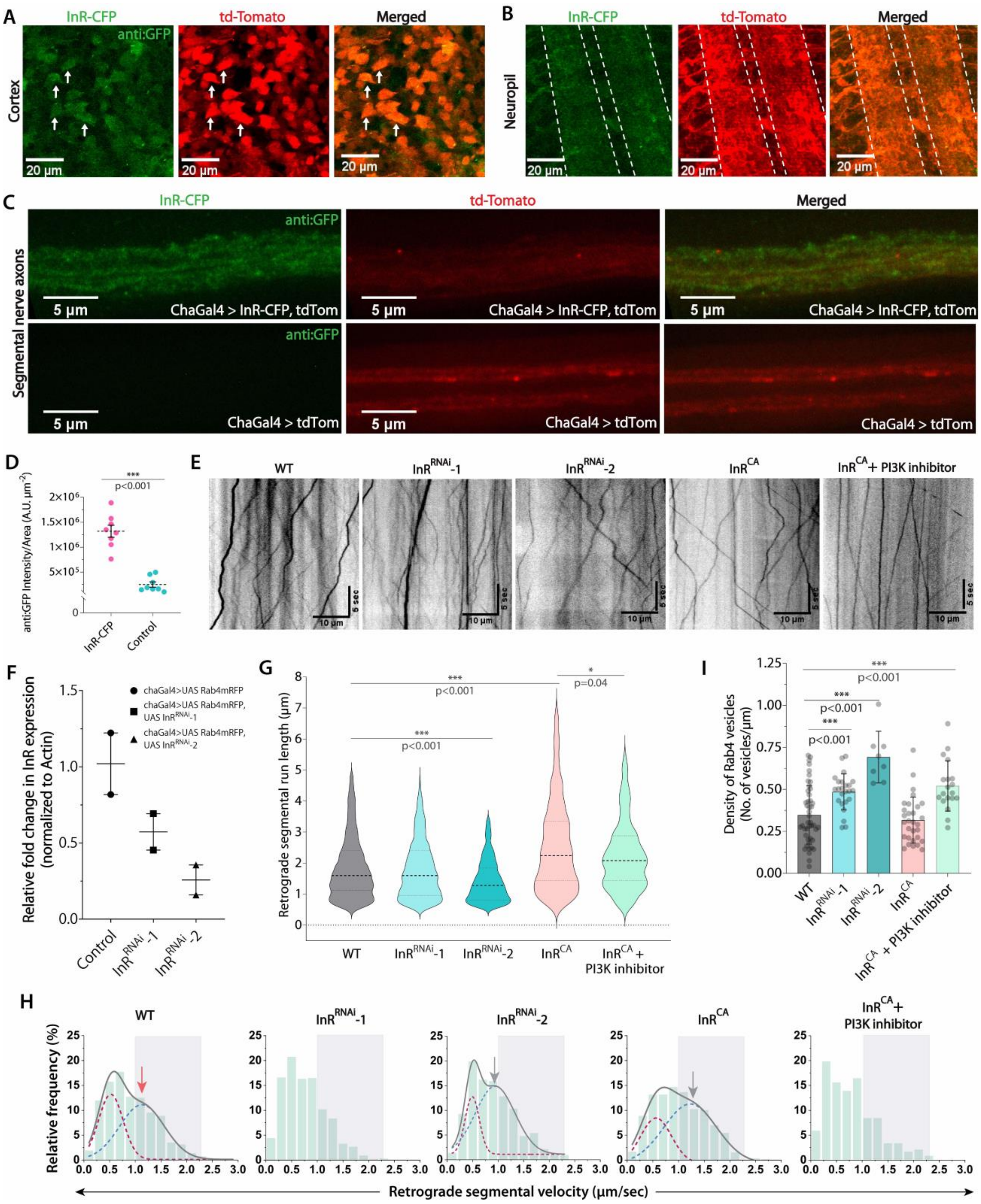

Journal of Cell Science • Supplementary information

Fig. S3. InR-mediated signaling contributes to the axonal transport of Rab4 vesicles. (A-C) Representative images of localizations of InRCFP (green), expressed using chaGal4 and stained using anti-GFP, in the cortex (A), synaptic region (B), and segmental nerve (C) of the VNC. UAS-tdTomato was coexpressed to mark the cholinergic neurons. **(D)** Quantification of InRCFP staining (anti-GFP) in the segmental nerves. **(E)** Representative kymographs depicting Rab4 vesicle transport upon knockdown of endogenous InR and overexpression of InR^{CA} (constitutively active mutant of insulin receptor) in cholinergic neurons. **(F)** Relative fold change in InR mRNA levels in isolated larval brains from *cha>InR*^{RNAi}*-1* and *cha>InR*^{RNAi}*-2* larvae. Values are normalized to Actin mRNA and each bar represents average of two trials and the whiskers represent the range. **(G-I)** Retrograde segmental run length (G), and retrograde segmental velocity (H), and density of Rab4 vesicles (I) in the wild-type control (WT) and different InR^{RNAi} and InR^{CA} overexpression backgrounds in distal cholinergic axons at 90 hours AEL (n>200 runs, N = 3-5 larvae each). Note: WT data column (I) is the same as 90hours AEL/WT data presented in Fig.S2D, Fig.S4F, Fig. S5B, Fig. S6B, Fig.S8D, and Fig. S9B. The cumulative distribution (grey) as a sum of two Gaussians (maroon and blue dotted lines) highlights slow (maroon) and fast-moving (blue) populations (See methods for details). Grey box marks the fast-moving (≥1.5μm/sec) runs. The pairwise significance of difference was estimated using the Mann-Whitney U-test**.**

Journal of Cell Science • Supplementary information

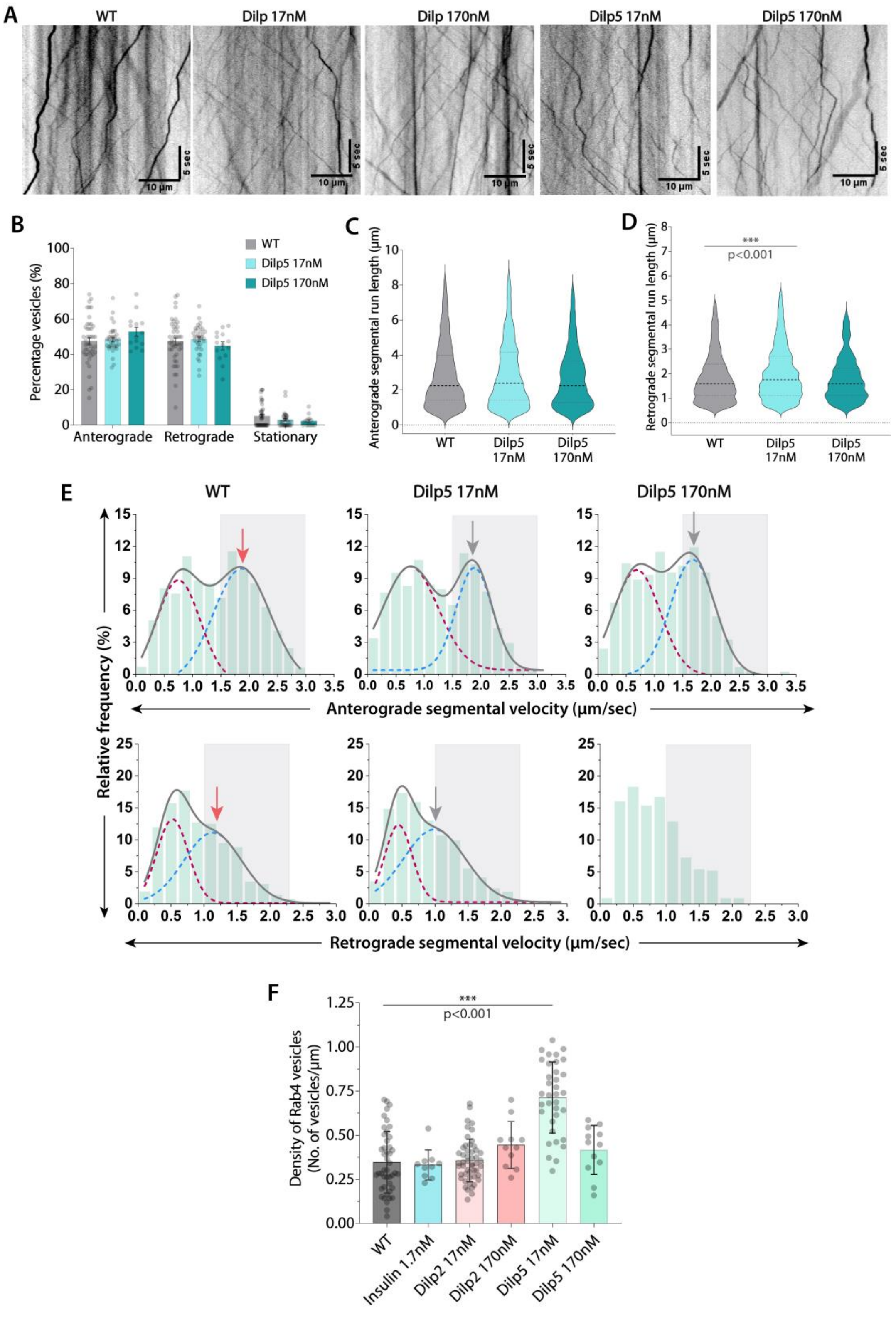

Journal of Cell Science • Supplementary information

Fig. S4. Acute Dilp5 stimulation does not increase the anterograde fraction, velocity, and run length of Rab4 vesicles in the axons. (A) Representative kymographs depicting Rab4 vesicle transport upon acute treatment (15 mins) with Dilp2 and Dilp5 (17nM and 170nM). Relative distribution of the Rab4 vesicle movement (n≥14 segmental nerves, N = 3-5 larvae) in the wild-type control without insulin (WT) and in the presence of different concentrations of *Drosophila* insulin-like peptide (Dilp5) at 90 hours AEL. The pairwise significance of difference was estimated using the Mann-Whitney U-test. **(B, C)** Anterograde (B) and retrograde (C) segmental run length (µm) of Rab4 vesicles in wild-type control (WT) and different treatment backgrounds (n>800 runs, N=3-5 larvae each). The pairwise significance of difference was estimated using the Mann-Whitney U-test. **(D)** Anterograde (top row) and retrograde (bottom row) segmental velocity distributions of Rab4 vesicles in wild-type control (WT) and different treatment backgrounds (n>800 runs; N=3-5 larvae). The cumulative distribution (grey) as a sum of two Gaussians (maroon and blue dotted lines) to highlight slow (maroon) and fast-moving (blue) populations (See methods for details). Grey box marks the fast-moving runs (≥1.5 µm/sec for anterograde and ≥1.0 µm/sec for retrograde). **(E)** Density of Rab4 vesicles (No. of vesicles/µm) in the distal axons of cholinergic neurons upon different pharmacological treatments for 15mins. Note: WT data column is the same as 90hours AEL/WT data presented in Fig.S2D, Fig.S3I, Fig. S5B, Fig. S6B, Fig.S8D, and Fig. S9B.The pairwise significance of difference was estimated using the Mann-Whitney U-test.

Journal of Cell Science • Supplementary information

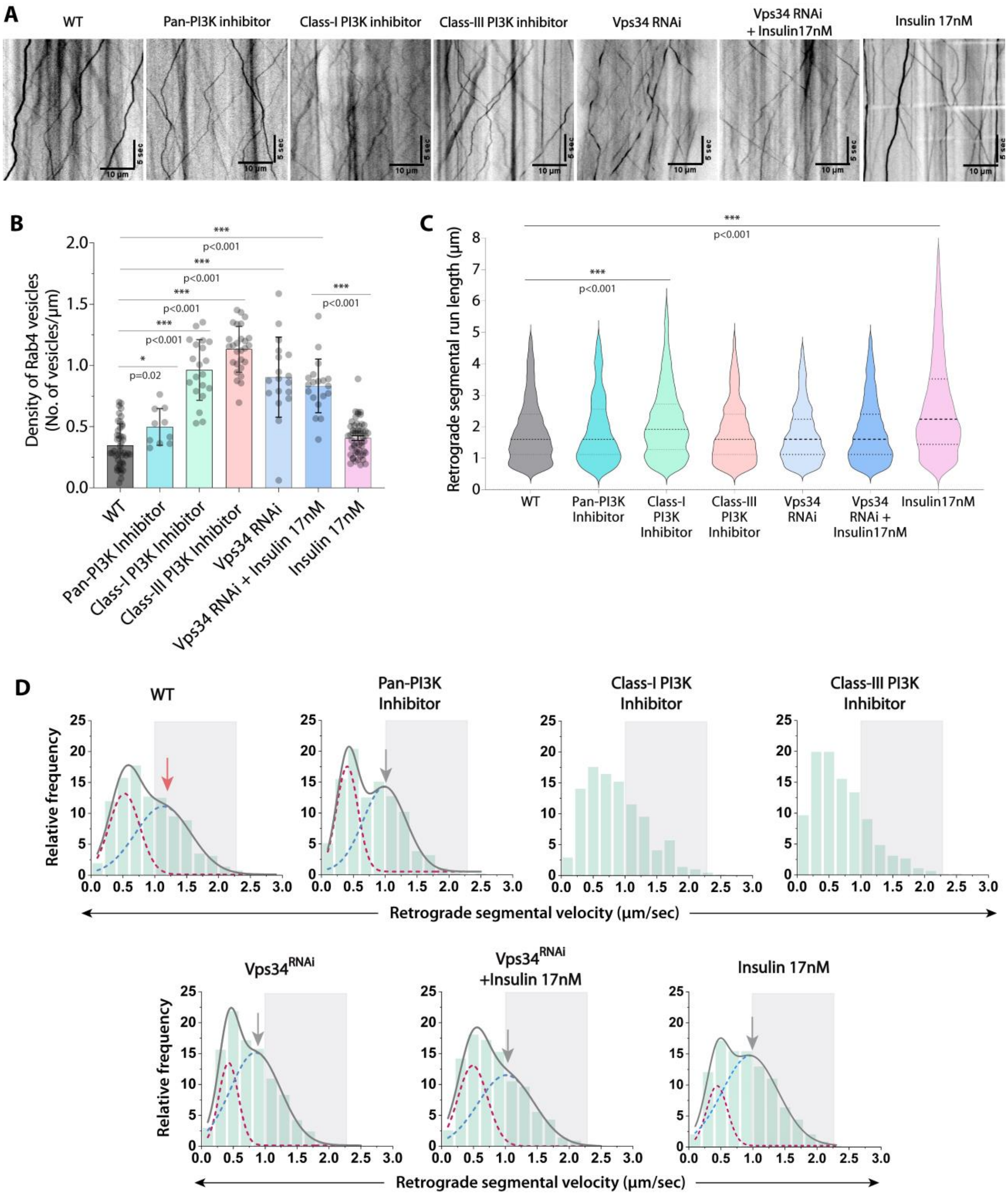

Fig. S5. Effects of acute inhibition and knockdown of Class-III PI3K/Vps34 on retrograde movement parameters of Rab4 vesicles in the axon. (A) Representative kymographs depicting transport of Rab4 vesicles in different pharmacological and

Journal of Cell Science • Supplementary information

genetic perturbation backgrounds. **(B)** Density of Rab4 vesicles (No. of vesicles/µm) in the distal axons of cholinergic neurons upon different pharmacological treatments for 15mins and genetic perturbations. Note: WT data column is the same as 90hours AEL/WT data presented in Fig.S2D, Fig.S3I, Fig. S4F, Fig. S6B, Fig.S8D, and Fig. S9B. The pairwise significance of difference was estimated using the Mann-Whitney U-test. **(C-D)** Retrograde segmental run length (C) and retrograde segmental velocity distributions (D) of Rab4 vesicles in the wild-type control (WT), in the presence of different class-specific PI3K inhibitors, and Vps34RNAi backgrounds in the absence and presence of insulin at 90 hours AEL (n>300 runs, N=3-5 larvae each). The cumulative distribution (grey) as a sum of two Gaussians (maroon and blue dotted lines) highlights slow (maroon) and fast-moving (blue) populations (See methods for details). Grey box marks the fast-moving (≥ 1.0 µm/sec) runs. The pairwise significance of difference was estimated using the Mann-Whitney U-test.

Journal of Cell Science • Supplementary information

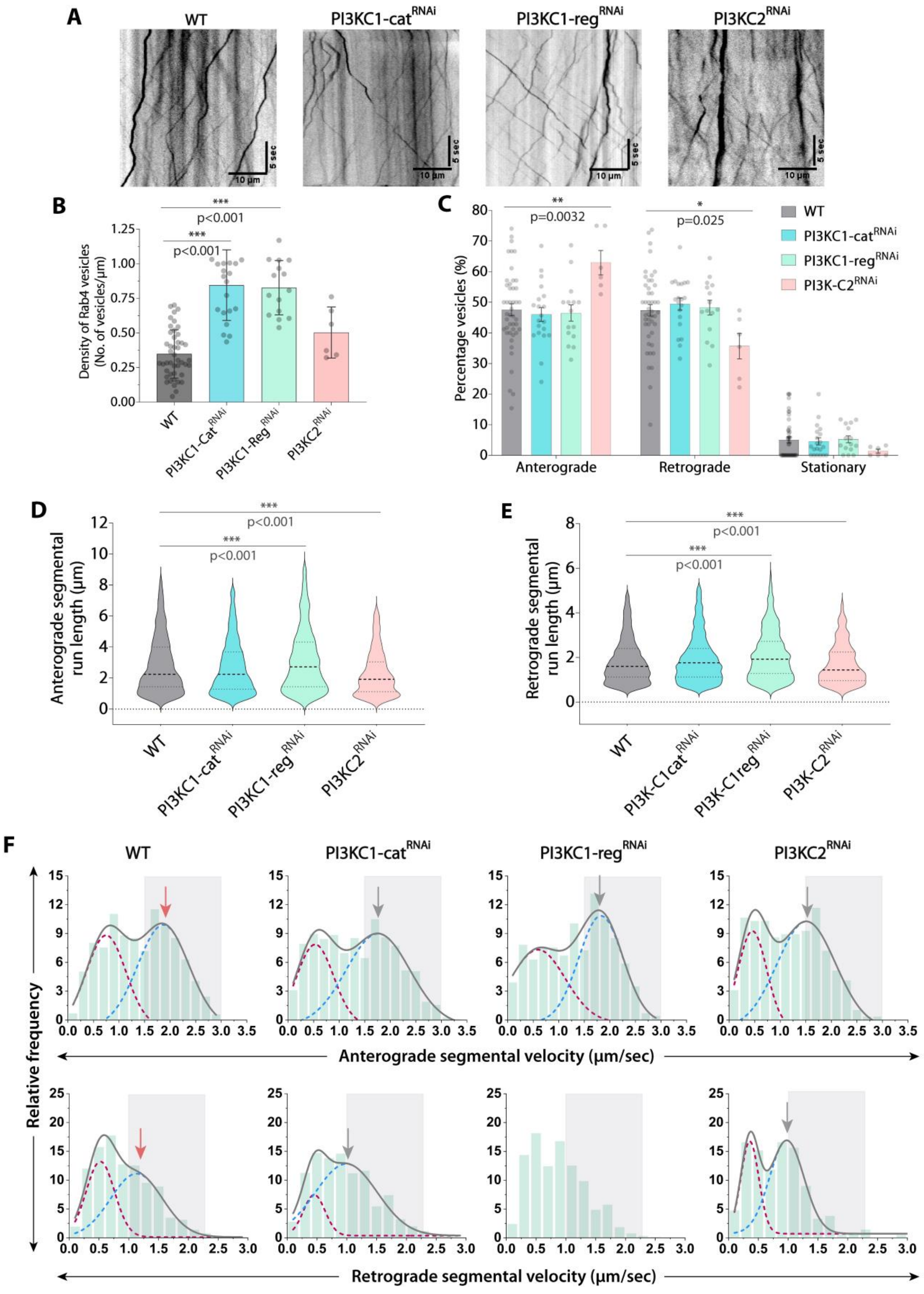

Journal of Cell Science • Supplementary information

Fig. S6. Cell-specific knockdown of Class-I and Class-II PI3Ks does not reduce anterograde fraction of Rab4 vesicles in the axons. (A) Representative kymographs depicting Rab4 vesicle transport upon knockdown of Class-I and Class-II PI3K in cholinergic neurons. **(B)** Density of Rab4 vesicles (No. of vesicles/µm) in the distal axons of cholinergic neurons upon knockdown of Class-I and Class-II PI3K in cholinergic neurons of third instar *Drosophila* larvae. Note: WT data column is the same as 90hours AEL/WT data presented in Fig.S2D, Fig.S3I, Fig. S4F, Fig. S5B, Fig.S8D, and Fig. S9B. The pairwise significance of difference was estimated using the Mann-Whitney U-test. **(C)** Relative distribution of the Rab4 vesicle movement in the wild-type control (WT) and different class-specific $PI3K^{RNAi}$ backgrounds at 90 hours AEL (n>6 segmental nerves, N=3-5 larvae each). The pairwise significance of difference was estimated using the Mann-Whitney U-test. **(D, E)** Anterograde (B) and retrograde (C) segmental run length (µm) of Rab4 vesicles in the wild-type control (WT) and different class-specific $PI3K^{RNAi}$ backgrounds (n>200 runs, N=3-5 larvae each). The pairwise significance of difference was estimated using the Mann-Whitney U-test. **(F)** Anterograde (top row) and retrograde (bottom row) segmental velocity distributions of Rab4 vesicles in the wild-type control (WT) and class-specific $PI3K^{RNAi}$ knockdown backgrounds (n>200 runs, N=3-5 larvae each).The cumulative distribution (grey) as a sum of two Gaussians (maroon and blue dotted lines) to highlight slow (maroon) and fast-moving (blue) populations (See methods for details). Grey box marks the fast-moving runs (≥1.5 µm/sec for anterograde and ≥1.0 µm/sec for retrograde).

Journal of Cell Science • Supplementary information

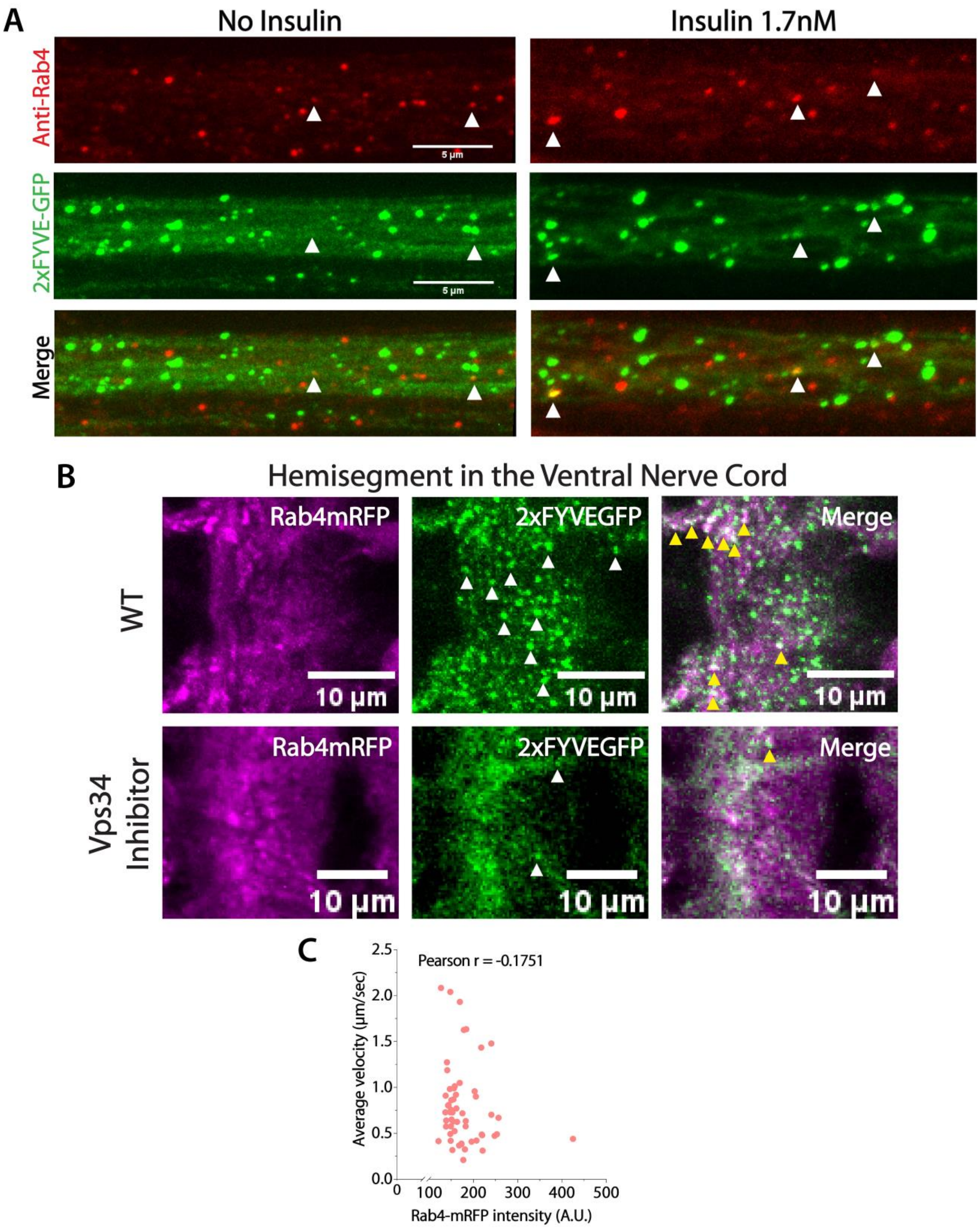

Journal of Cell Science • Supplementary information

Fig. S7. (A) Colocalization of endogenous Rab4 on 2xFYVEGFP vesicles in cholinergic axons. The frequency of colocalization increases from ~3% to ~8% (>150 vesicles sampled from >5 ROIs from N=2-3 animals) upon acute stimulation with 1.7nM insulin. Arrowheads mark Rab4-2xFYVEGFP colocalized vesicles. Here, one should note that the Rab4 antibody will mark all Rab4 vesicles and soluble Rab4 aggregates in all axons (sensory and motor neurons) and in the cytoplasm of the glial cells surrounding the axons. Whereas the *cha>2xFYVE-GFP* expression will mark PI(3)P on vesicles inside cholinergic axons only. Hence, the larger base effect will dilute the actual frequency of colocalization in cholinergic neuron. Notwithstanding the difference in baseline values, we observed >2 folds increase in the colocalization with endogenous Rab4, as observed earlier in the *cha>Rab4mRFP, 2xFYVE-GFP* background after insulin stimulation (Fig 6D). Therefore, these results also confirmed that Rab4 vesicles are indeed present in cholinergic axons. **(B)** One hemisegment of third-instar larval VNC highlighting the effect of acute treatment with Vps34 inhibitor on PI(3)P localization in the synaptic region of VNC. White arrowheads mark the PI(3)P puncta and yellow arrowheads mark Rab4mRFP-2xFYVEGFP colocalized puncta. A visible reduction was observed after the treatment with Vps34 inhibitor. **(C)** Correlation plot between Rab4 vesicle intensity and velocity in the *cha>Rab4mRFP* background. Note that the Pearsons r value is very similar to the intensity vs velocity correlation obtained in the *cha>Rab4mRFP, 2xFYVEGFP* background (0.1751 vs 0.1735) ruling out any influence of 2xFYVEGFP overexpression on the correlation between intensity of individual Rab4 vesicles and their corresponding velocity values.

Journal of Cell Science • Supplementary information

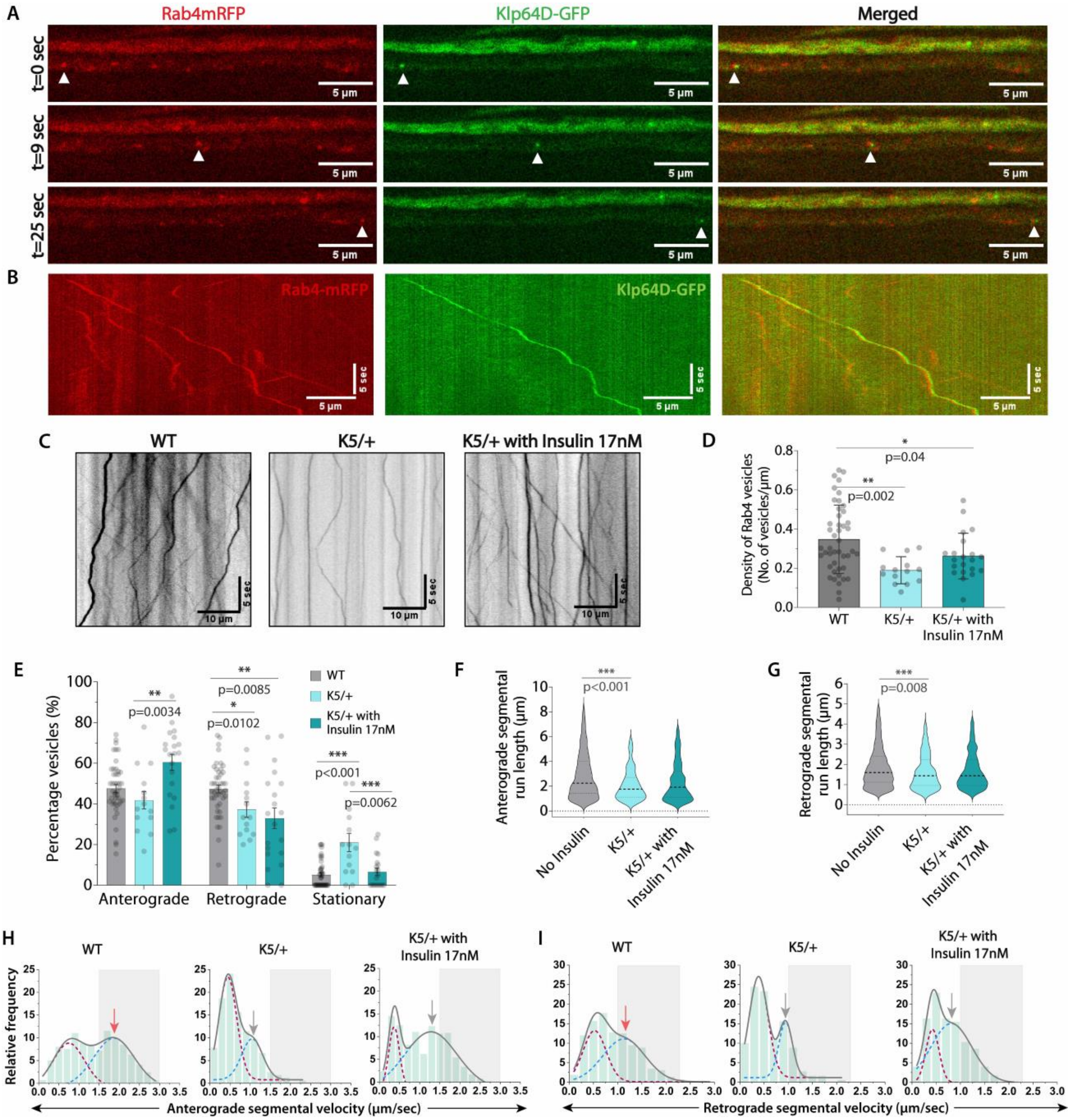

Fig. S8. Kinesin-2 does not regulate the increase in anterograde fraction of Rab4 vesicles in the axons downstream of insulin signaling. (A, B) Simultaneously acquired dual-channel time-lapse images of segmental nerve (A) and kymographs (B) depict comigration of genetically-encoded Rab4mRFP (red) and Klp64DGFP (green, KIF3A ortholog) in cholinergic axons. **(C)** Representative kymographs depicting axonal transport of Rab4 vesicles in K5 heterozygous mutant backgrounds. **(D)** Density of Rab4 vesicles (No. of vesicles/µm) in the distal axons of cholinergic neurons in K5

heterozygous mutant backgrounds. Note: WT data column is the same as 90hours AEL/WT data presented in Fig.S2D, Fig.S3I, Fig. S4F, Fig. S5B, Fig.S6B, and Fig. S9B. The pairwise significance of difference was estimated using the Mann-Whitney U-test. **(E)** Relative distribution of the Rab4 vesicle movement in the wild-type control (WT) and kinesin-2 mutant, K5, in the heterozygous background in the absence and presence of insulin at 90 hours AEL (n≥14 segmental nerves, N=3-5 larvae each). The pairwise significance of difference was estimated using the Mann-Whitney U-test. **(F, G)** Anterograde (F) and retrograde (G) segmental run length (µm) of Rab4 vesicles in the wild-type control (WT) and different genetic backgrounds (n>150 runs, N=3-5 larvae each). The pairwise significance of difference was estimated using the Mann-Whitney U-test. **(H, I)** Anterograde (H) and retrograde (I) segmental velocity distributions of Rab4 vesicles in the wild-type control (WT) and different genetic backgrounds (n>150 runs, N=3-5 larvae each) at 90 hours AEL. The cumulative distribution (grey) as a sum of two Gaussians (maroon and blue dotted lines) to highlight slow (maroon) and fast-moving (blue) populations (See methods for details). Grey box marks the fast-moving runs (≥1.5 µm/sec for anterograde and ≥1.0 µm/sec for retrograde).

Journal of Cell Science • Supplementary information

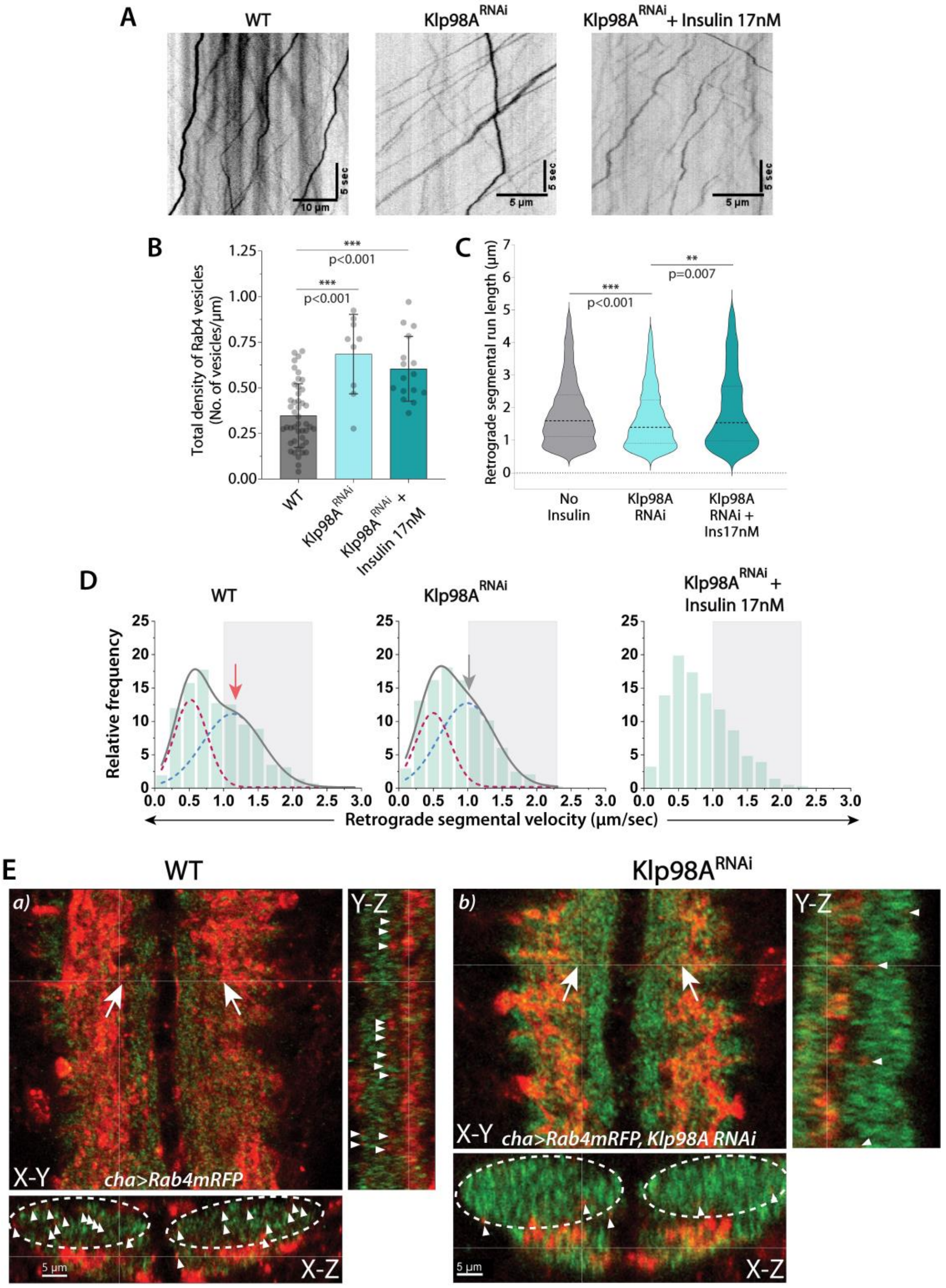

Fig. S9. Effect of Klp98A knockdown on the retrograde movement parameters of Rab4 vesicles in the axons. (A) Representative kymographs depicting axonal transport of Rab4 vesicles upon knockdown of Klp98A motor in cholinergic neurons. **(B)** Density of Rab4 vesicles (No. of vesicles/µm) in the distal axons of cholinergic neurons

Journal of Cell Science • Supplementary information

in Klp98A RNAi backgrounds. Note: WT data column is the same as 90hours AEL/WT data presented in Fig.S2D, Fig.S3I, Fig. S4F, Fig. S5B, Fig.S8D, and Fig. S8D. The pairwise significance of difference was estimated using the Mann-Whitney U-test. **(C, D)** Retrograde segmental run length (A) and retrograde segmental velocity distributions (B) of Rab4 vesicles in the wild-type control (WT) and Klp98A^{RNAi} in the absence and presence of insulin (n>400 runs, N=4-6 larvae each) at 90 hours AEL. The cumulative distribution (grey) as a sum of two Gaussians (maroon and blue dotted lines) highlights slow (maroon) and fast-moving (blue) populations (See methods for details). Grey box marks the fast-moving (≥ 1.0 μm/sec) runs. The pairwise significance of difference was estimated using the Mann-Whitney U-test. **(E)** Optical sections of XY, YZ, and XZ planes of the A3-A6 segments of 80 hours AEL larval VNCs from wildtype control **(a)** and Klp98A RNAi **(b)** backgrounds, respectively, expressing *cha>Rab4mRFP* (red) and stained with Anti-Brp (green). The sections were obtained from deconvoluted 3D reconstruction of confocal image stacks of the specimens using Imaris software. X-Y section is obtained from the middle part of the dorsoventral axis, which consists of prominent ipsilateral projections of the proprioceptive and cholinergic sensory neurons (1). The neuropil regions in X-Z sections (marked by dotted ellipses) depict cholinergic projections marked by Rab4mRFP (arrows in X-Y sections). Note several Rab4mRFP-marked boutons (arrowheads) are juxtaposed with one or more Brp puncta in WT background **(a)**. Notably, the Brp puncta are visibly more numerous than the RabmRFP boutons in the Klp98A RNAi background **(b)**. This result is consistent with the quantification of Brp and Rab4mRFP enrichment in the synaptic regions of VNCs as shown in Fig 8G. This evidence also highlighted the presence of Rab4 in the synaptic regions marked by Brp. Further, we observed that neurites strongly marked with Rab4mRFP had relatively fewer Brp punta (arrows). This mutual exclusion is also consistent with the proposal that the presence of Rab4 at the presynaptic terminal may suppress synapse formation as discussed in the text.

Journal of Cell Science • Supplementary information

Table S1. Percentage of fast- and slow-moving vesicles in anterograde and retrograde directions across conditions, pharmacological treatments, and genetic perturbations.

Condition/Treatment/Perturbation	Anterograde %		Retrograde %	
	0-1.6μm/sec	>1.6μm/sec	0-1μm/sec	>1μm/sec
72 hours AEL (After Egg Laying)	76.92	23.08	73.46	26.54
80 hours AEL	59.84	40.16	68.59	31.41
No insulin/90 hours AEL	56.82	43.18	60.05	39.95
Insulin 1.7nM	48.33	51.67	50.53	49.47
Dilp2 17nM	51.34	48.66	59.98	40.02
Dilp2 170nM	41.46	58.54	47.84	52.16
Dilp5 17nM	62.32	37.68	64.63	35.37
Dilp5 170nM	68.83	31.17	67.42	32.58
LY294002 50uM	78.61	21.39	68.57	31.43
HS173 50nM	64.07	35.93	66.15	33.85
SAR405 50uM	81.44	18.56	78.38	21.62
InR^{CA}	42.65	57.35	51.54	48.46
InR^{CA} + LY294002 560uM	70.2	29.8	71.73	28.27
InR^{RNAi}-1	72.94	27.06	74.4	25.6
InR^{RNAi}-2	72.56	27.44	62.66	37.34
Class-I PI3K cat^{RNAi}	59.16	40.84	57.08	42.92
Class-I PI3K reg^{RNAi}	57.13	42.87	64.04	35.96
Class-II PI3K^{RNAi}	70.11	29.89	64.93	35.07
Vps34^{RNAi}	67.52	32.48	73.31	26.69
Vps34^{RNAi} + Insulin 17nM	63.66	36.34	67.29	32.71
Klp64D^{K5}	96.38	3.62	84.44	15.56
Klp64D^{K5} + Insulin 17nM	77.8	22.2	75.07	24.93
Klp98a^{RNAi}	84.44	15.56	66.41	33.59
Klp98a^{RNAi} + Insulin 17nM	73.72	26.28	68.55	31.45

Table S2. Raw data values for volume and intensity of Brp per hemisegment and segment-wise in A3-A6 from 72-90 hours AEL (Fig.1 D-E and fig. S1B-D) is organized in different sheets in the spreadsheet.

Available for download at
https://journals.biologists.com/jcs/article-lookup/doi/10.1242/jcs.264782#supplementary-data

Table S3. Raw data values for volume and intensity of soluble GFP marked by *chaGal4* per hemisegment in A3-A6 (Fig.1 F-H) is organized in different sheets in the spreadsheet.

Available for download at
https://journals.biologists.com/jcs/article-lookup/doi/10.1242/jcs.264782#supplementary-data

Table S4. Raw data values for density (intensity per unit volume) of Rab4 and Bruchpilot per hemisegment in A3-A6 (Fig. 2B).

Available for download at
https://journals.biologists.com/jcs/article-lookup/doi/10.1242/jcs.264782#supplementary-data

Table S5. Raw data values of percentage of Rab4 vesicles in the anterograde, retrograde, and stationary categories (fraction) at different developmental timepoints, in different conditions, or after a pharmacological or genetic perturbation (Fig. 2E, 3A, 4A, 5A, 5D, 7C and fig. S4B, S6C, S8E).

Available for download at
https://journals.biologists.com/jcs/article-lookup/doi/10.1242/jcs.264782#supplementary-data

Table S6. Raw data values of segmental run length (µm) of Rab4 vesicles in the anterograde and retrograde direction at different developmental timepoints, in different conditions, or after a pharmacological or genetic perturbation (Fig. 2F, S2A, 3B, 4B-C, 5B, E, 7D and fig. S3G, S4C-D, S5C, S6D-E, S8F-G, and S9C).

Available for download at
https://journals.biologists.com/jcs/article-lookup/doi/10.1242/jcs.264782#supplementary-data

Table S7. Raw data values of segmental velocity (µm/sec) of Rab4 vesicles in the anterograde and retrograde direction at different developmental timepoints, in different conditions, or after a pharmacological or genetic perturbation (Fig. 2G, S2B, 3C, S3H, 4D, S4E, S5D, S5B, S6F, S8H-I, and S9D).

Available for download at
https://journals.biologists.com/jcs/article-lookup/doi/10.1242/jcs.264782#supplementary-data

Table S8. Raw data values of anti-GFP intensity per unit area values (A.U. μm^{-2}) in the segmental nerve axons for control and InR-CFP overexpression background (fig. S3C-D).

Available for download at
https://journals.biologists.com/jcs/article-lookup/doi/10.1242/jcs.264782#supplementary-data

Table S9. Sheet 1: Raw data values of *percentage colocalized vesicles* of the total (Rab4mRFPand 2xFYVE-GFP) in different pharmacological treatments in wandering third instar larvae (Fig. 6C-D)and at different developmental timepoints (Fig. 8C-D). Sheet 2: Raw data values of *total Rab4vesicles and colocalized vesicle numbers* in different pharmacological treatments and at different developmental time points.

Available for download at
https://journals.biologists.com/jcs/article-lookup/doi/10.1242/jcs.264782#supplementary-data

Table S10. Sheet 1: Raw data values of Rab4mRFP and 2xFYVEGFP intensities (A.U.) on single vesicles and their average velocities (µm/sec) in the distal axons of cholinergic neurons from the genotype *cha>Rab4mRFP, 2xFYVEGFP* (Fig. 6E-F). Sheet 2: Raw data values of Rab4mRFP intensities (A.U.) on single vesicles and their average velocities (µm/sec) in the distal neurons from the genotype *cha>Rab4mRFP/+* (Fig. S7C).

Available for download at
https://journals.biologists.com/jcs/article-lookup/doi/10.1242/jcs.264782#supplementary-data

Table S11. Sheet 1: Raw data values of *percentage colocalized vesicles* of the total (Rab4mRFP and Klp98A-GFP) in different pharmacological treatments in wandering third instar larvae (Fig. 7F-G) and at different developmental timepoints (Fig. 8A-B). Sheet 2: Raw data values of *total Rab4 vesicles and Rab4mRFP-Klp98AGFP colocalized vesicle numbers* in different pharmacological treatments and at different developmental time points.

Available for download at
https://journals.biologists.com/jcs/article-lookup/doi/10.1242/jcs.264782#supplementary-data

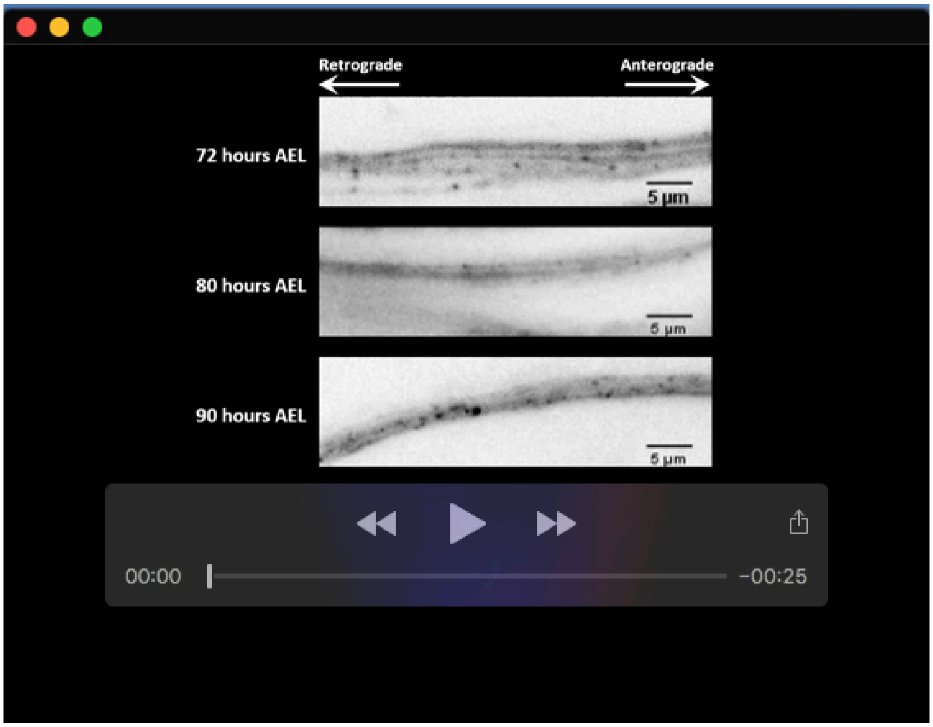

Movie 1. Axonal transport of Rab4mRFP vesicles in the distal axons of *Drosophila* cholinergic neurons at 72, 80, and 90 hours AEL. Widefield time-lapse imaging was performed at 10fps, and the movies are played at 50fps. Duration of all the representative movies is 30sec.

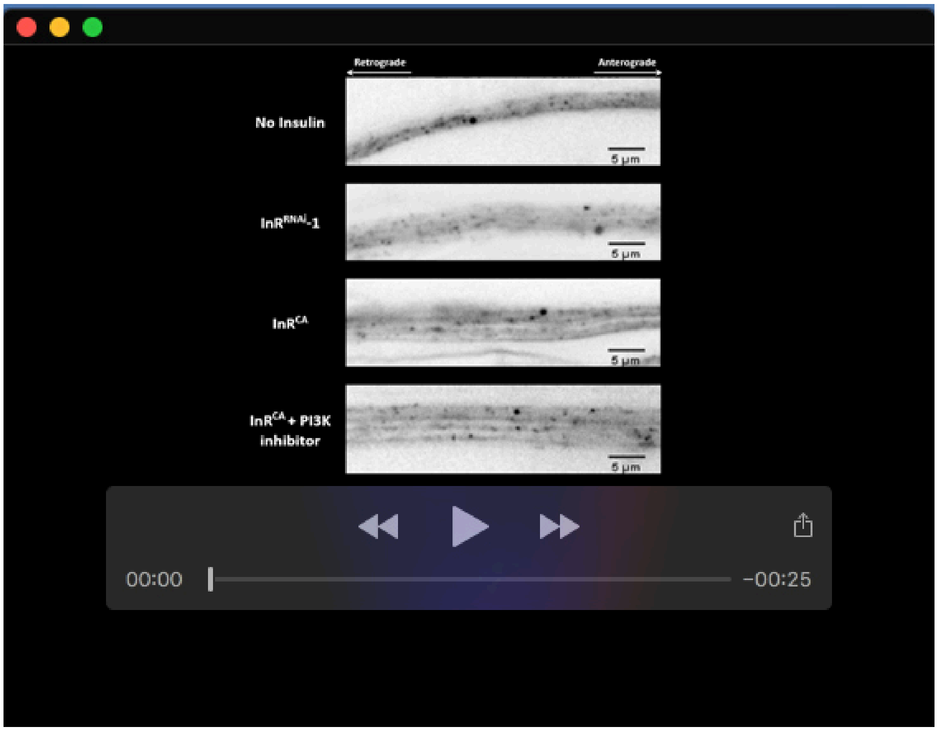

Movie 2. Effect of various genetic and pharmacological perturbations of insulin signalling on the axonal transport of Rab4mRFP vesicles in the distal axons of *Drosophila* cholinergic neurons at 90 hours AEL. Widefield time-lapse imaging was performed at 10fps, and the movies are played at 50fps. Duration of all the representative movies is 30sec.

Journal of Cell Science • Supplementary information

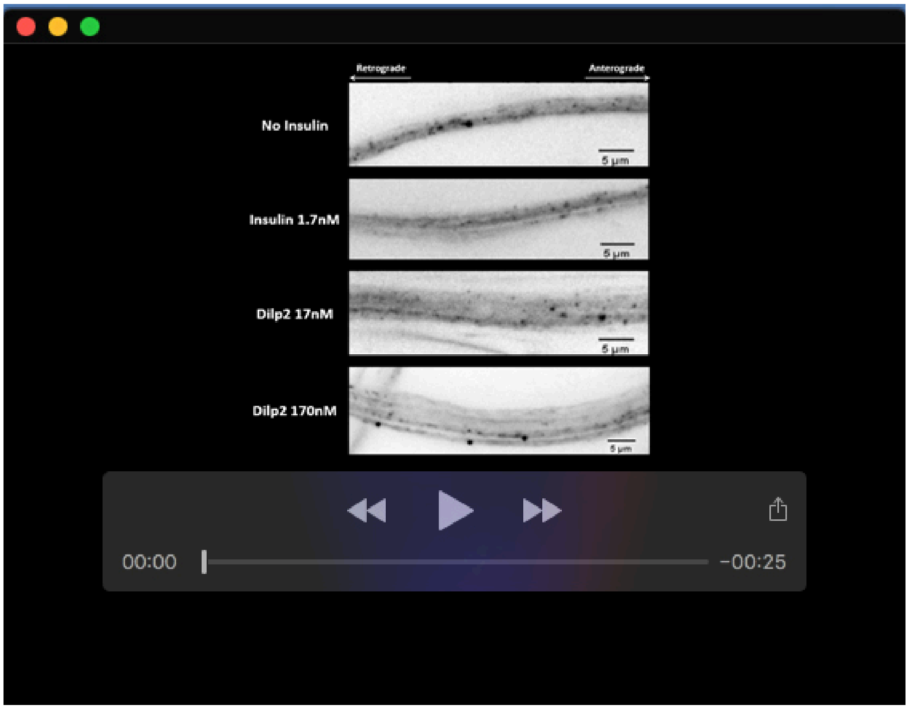

Movie 3. Effect of acute stimulation of human insulin and Dilp2 on the axonal transport of Rab4mRFP vesicles in the distal axons of *Drosophila* cholinergic neurons at 90 hours AEL. Widefield time-lapse imaging was performed at 10fps, and the movies are played at 50fps. Duration of all the representative movies is 30sec.

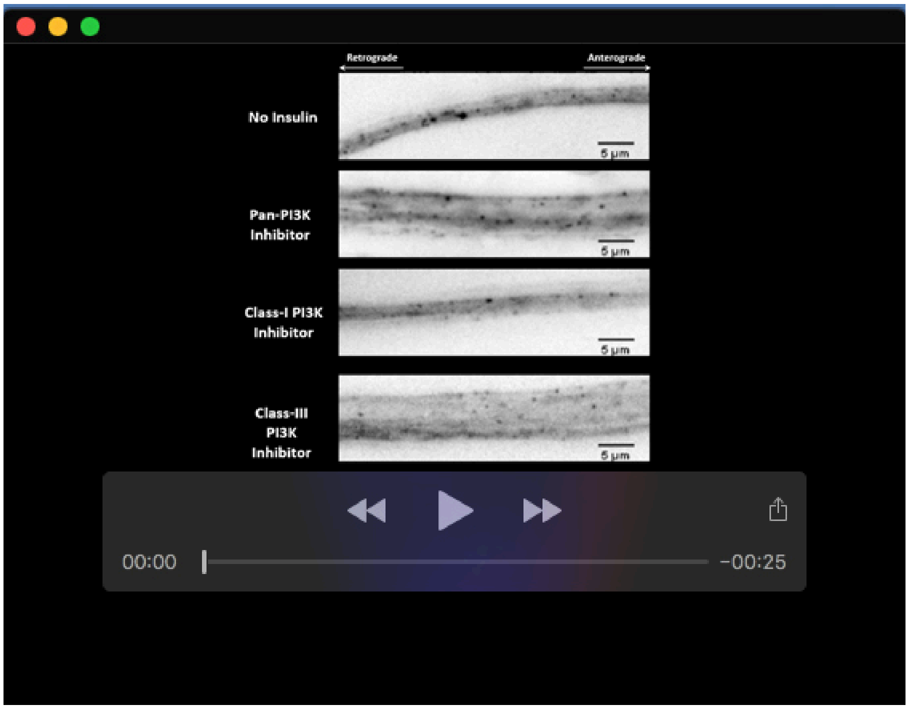

Movie 4. Effect of acute inhibition of PI3Kinases on the axonal transport of Rab4mRFP vesicles in the distal axons of *Drosophila* cholinergic neurons at 90 hours AEL. Widefield time-lapse imaging was performed at 10fps, and the movies are played at 50fps. The duration of all the representative movies is 30sec.

Journal of Cell Science • Supplementary information

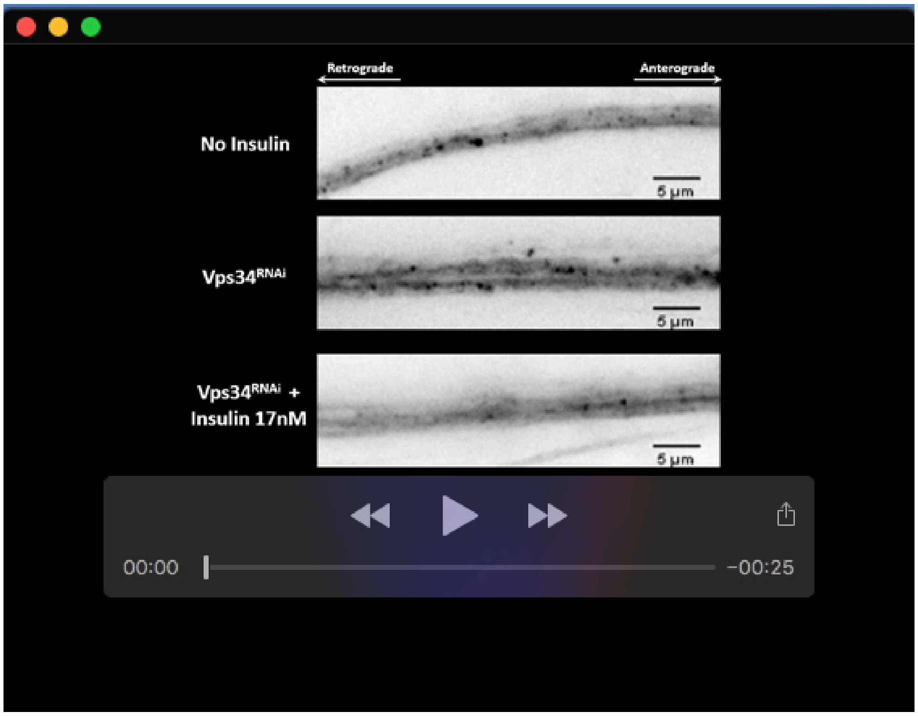

Movie 5. Effect of cell-specific knockdown of Class-III PI3K/Vps34 with and without acute insulin stimulation on the axonal transport of Rab4mRFP vesicles in the distal axons of *Drosophila* cholinergic neurons at 90 hours AEL. Widefield time-lapse imaging was performed at 10fps, and the movies are played at 50fps. The duration of all the representative movies is 30sec.

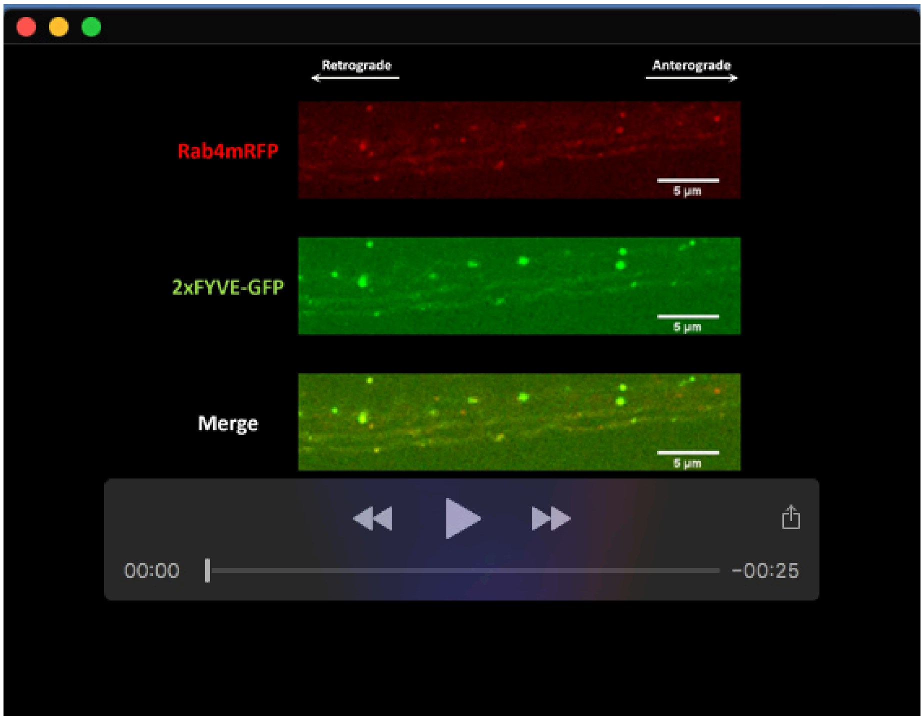

Movie 6. Simultaneous dual-color time-lapse imaging performed using Spinning-disc confocal microscopy shows comigration of genetically-encoded PI(3)P biosensor (2xFYVEGFP) and Rab4mRFP in the distal axons of *Drosophila* cholinergic neurons. Data was collected at 8-9fps, and movies are played at 50fps. The duration of the representative movie is 20sec.

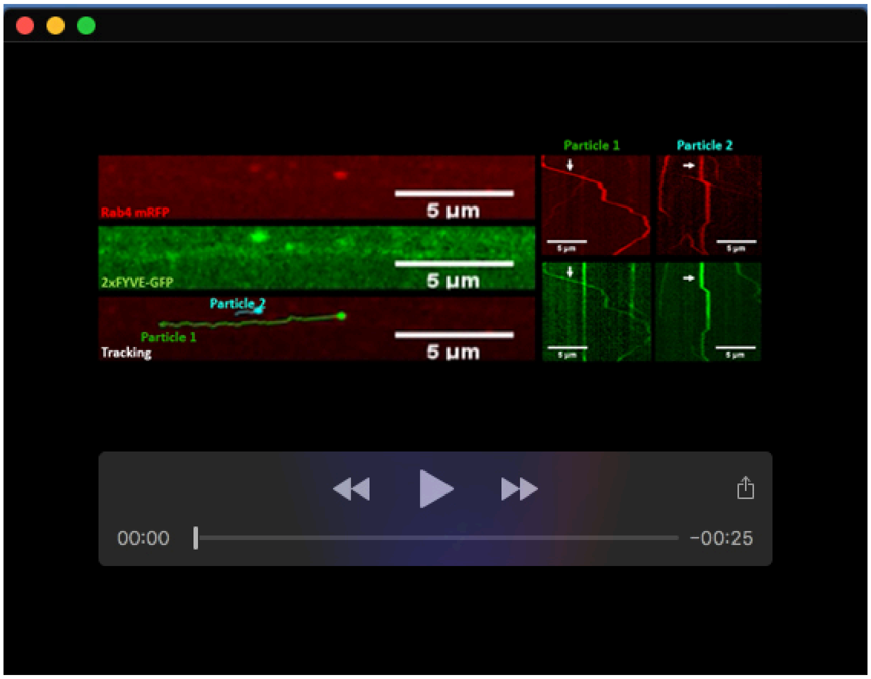

Movie 7. Simultaneous dual-color time-lapse imaging performed using Spinning-disc confocal microscopy shows comigration of 2xFYVEGFP and Rab4mRFP in the distal axons of *Drosophila* cholinergic neurons. Particle 1 (green) and Particle 2 (cyan) are representative examples of particles with a low and high 2xFYVEGFP/Rab4mRFP intensity ratio (as plotted in Figure 6F), respectively. Data was collected at 8-9fps, and movies are played at 50fps. The duration of the representative movie is 20sec.

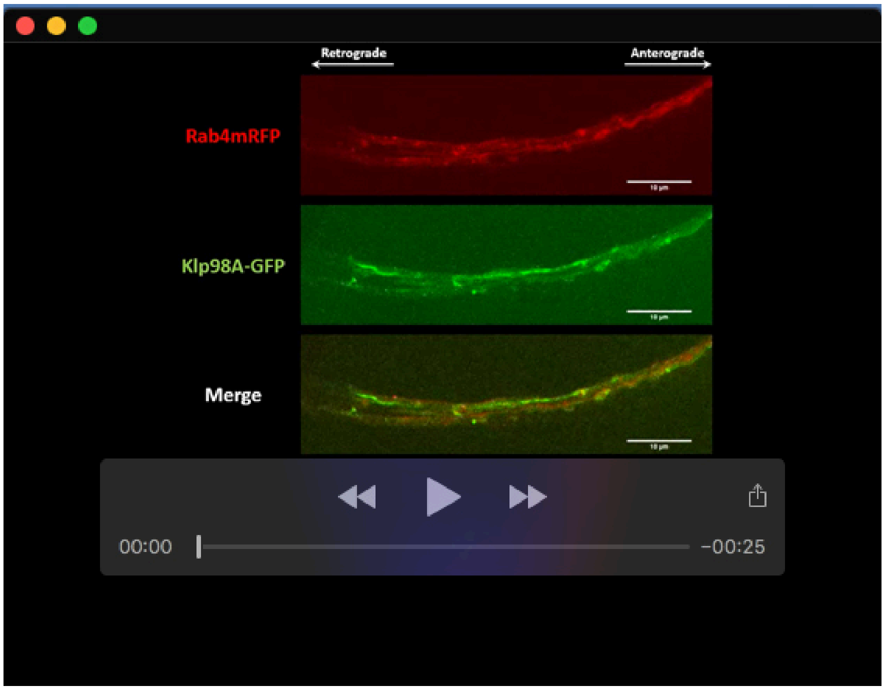

Movie 8. Simultaneous dual-color time-lapse imaging performed using Spinning-disc confocal microscopy shows comigration of Klp98AGFP and Rab4mRFP in the distal axons of *Drosophila* cholinergic neurons. Data was collected at 8-9fps, and movies are played at 50fps. The duration of the representative movie is ~20sec.

Journal of Cell Science • Supplementary information
